# Supplementary material for: The relationship between sleep duration and fruit/vegetable intakes in UK adults: a cross-sectional study from the National Diet and Nutrition Survey
Source: BMJ Open. 2018 Apr 27;8(4):e020810. doi: 10.1136/bmjopen-2017-020810 (PMC5922510; doi:10.1136/bmjopen-2017-020810)
Supplement: Supplementary file 1 [file bmjopen-2017-020810supp001.pdf]

Online supplementary material

Table 1. The association between sleep duration categories and FV intakes and associated biomarkers for adults from the NDNS years 1-4 after excluding participants who consume prescribed medicines.

		Sleep categories compared to the reference group (7-8 hours/day)			
**Model 2 (n=1044)**		**Short sleepers <7 hours/day**		**Long sleepers >8 hours/day**	
**FV intake**		**Mean difference (95%CI)**	**P value**	**Mean difference (95%CI)**	**P value**
Total fruit[(a)] (g/day)		-13 (-27, -0.4)	0.04	-17 (-34, -0.1)	0.04
Total veg[(b)] (g/day)		-10 (-24, 4)	0.1	-5 (-24, 2)	0.5
FV portions[(c)]		-0.2 (-0.5, -.005)	0.04	-0.1 (-0.5, 0.1)	0.3
5-a-day portions[(d)]		-0.2 (-0.5, -0.0003)	0.05	-0.1 (-0.5, 0.1)	0.3
Total FV [(e)](g/day)		-24 (-45,-2)	0.03	-23 (-51,4)	0.1
**Nutrients (mg)**					
Vitamin C diet only		-5 (-13, 2)	0.1	-4(-15,5)	0.3
Vitamin C *		-7 (-24, 10)	0.4	-11 (-33, 11)	0.3
**Biomarkers (μmol/l)**					
Vitamin C	n= 375	-0.6 (-5, 3)	0.7	5 (-0.09, 11)	0.05
Total caro[(f)]	n= 294	-0.2 (-0.5, -0.02)	0.03	-0.08 (-0.4, 0.2)	0.5
α-carotene	n= 378	-0.005 (-0.02,0.01)	0.5	-0.009 (-0.03, 0.01)	0.4
β-carotene	n= 402	-0.05 (-0.1, 0.02)	0.1	-0.02(-0.1, 0.07)	0.6
Lycopene	n= 403	-0.06 (-0.1, 0.01)	0.1	-0.06 (-0.1, 0.04)	0.2

566 participants reported taking prescribed medicines and were excluded from the analyses.

Table 2. The association between sleep duration categories and FV intakes and associated biomarkers for adults from the NDNS years 1-4 after excluding participants who reported consuming vitamins, minerals and/or supplements in the past year.

		Sleep categories compared to the reference group (7-8hours/day)			
**Model 2 (n=1084)**		**Short sleepers <7 hours/day**		**Long sleepers >8 hours/day**	
**FV intake**		**Mean difference (95%CI)**	**P value**	**Mean difference (95%CI)**	**P value**
Total fruit[(a)] (g/day)		-5 (-17,7)	0.4	-10 (-25, 0.5)	0.2
Total veg[(b)] (g/day)		-11 (-24, 1)	0.09	-7 (-24, 8)	0.3
FV portions[(c)]		-0.1 (-0.4, 0.07)	0.1	-0.1 (-0.4, 0.1)	0.3
5-a-day portions[(d)]		-0.1 (-0.4, 0.09)	0.2	-0.1 (-0.4, 0.2)	0.4
Total FV[(e)](g/day)		-16 (-36, 3)	0.1	-17 (-43,7)	0.1
**Nutrients (mg)**					
Vitamin C diet only		1 (-6, 8)	0.7	2 (-6, 11)	0.5
Vitamin C *		0.2 (-8, 8)	0.9	2 (-7, 13)	0.5
**Biomarkers (μmol/l)**					
Vitamin C	n= 462	-3 (-7, 0.6)	0.09	6 (1, 11)	0.009
Total caro [(f)]	n= 330	-0.2 (-0.4, -0.01)	0.03	0.06 (-0.2 ,0.3)	0.6
α-carotene	n= 461	-0.005 (-0.02,0.009)	0.4	0.0007 (-0.01, 0.02)	0.9
β-carotene	n= 492	-0.05 (-0.1, 0.002)	0.06	0.06 (-0.01,0.1)	0.1
Lycopene	n= 490	-0.08 (-0.1, -0.005)	0.03	-0.04 (-0.1, 0.04)	0.3

526 participants reported taking vitamins, minerals or supplements in the past year and were excluded from the analyses.

Table 3. The association between sleep duration categories and FV intakes and associated biomarkers for adults from the NDNS years 1-4 after excluding those who have a longstanding illness.

		**Sleep categories compared to the reference group (7-8hours)**			
**Model 2 (n= 1063)**		**Short sleepers <7 hours**		**Long sleepers >8 hours**	
**FV intake**		**Mean difference(95%CI)**	**P value**	**Mean difference (95%CI)**	**P value**
Total fruit[a] (g/day)		-15 (-29, -2)	0.02	-9 (-26, 7)	0.2
Total veg[b] (g/day)		-14 (-28, -0.04)	0.04	-10 (-28, 8)	0.2
FV portions[c]		-0.4 (-0.7, -0.1)	0.004	-0.1 (-0.5, 0.1)	0.2
5-a-day portions[d]		-0.4 (-0.7, -0.1)	0.005	-0.1 (-0.5 ,0.1)	0.3
Total FV[e](g/day)		-30 (-52, -8)	0.007	-20 (-48, 8)	0.1
**Nutrients (mg)**					
Vitamin C diet only		-5 (-14, 2)	0.1	-1 (-11, 9)	0.8
Vitamin C *		-12 (-30, 5)	0.1	-9 (-32, 14)	0.4
**Biomarkers (µmol/l)**					
Vitamin C	n= 474	-2 (-6, 1)	0.2	4 (-0.4 ,9)	0.07
Total caro [f]	n= 369	-0.3 (-0.5, -0.08)	0.007	-0.04 (-0.3,0.2)	0.7
α-carotene	n= 477	-0.006 (-0.02, 0.01)	0.4	0.001 (-0.01, 0.02)	0.9
β-carotene	n= 506	-0.06 (-0.1, -0.003)	0.03	0.01 (-0.06, 0.09)	0.6
Lycopene	n= 505	-0.07 (-0.1, 0.008)	0.08	-0.05 (-0.1, 0.05)	0.3

547 participants reported having a longstanding illness and were excluded from the analyses.

Table 4. The association between sleep duration categories and FV intakes and associated biomarkers for adults from the NDNS years 1-4 after excluding those who reported being vegetarian.

		Sleep categories compared to the reference group (7-8hours/day)			
**Model 2 (n= 1571)**		**Short sleepers <7 hours/day**		**Long sleepers >8 hours/day**	
**FV intake**		**Mean difference(95%CI)**	**P value**	**Mean difference (95%CI)**	**P value**
Total fruit[(a)] (g/day)		-14 (-25,-3)	0.009	-9 (-26,7)	0.2
Total veg[(b)] (g/day)		-11 (-22, 0.1)	0.05	-11 (-25,3)	0.1
FV portions[(c)]		-0.3 (-0.5, -0.07)	0.009	-0.3 (-0.5, -0.01)	0.03
5-a-day portions[(d)]		-0.3 (-0.5,-0.08)	0.008	-0.2 (-0.5, 0.008)	0.05
Total FV [(e)](g/day)		-25 (-43,-8)	0.004	-29 (-51, -7)	0.009
**Nutrients (mg)**					
Vitamin C diet only		-5 (-12, 0.9)	0.09	-4 (-12, 4)	0.3
Vitamin C *		-4 (-18, 8)	0.4	-8 (-25, 8)	0.3
**Biomarkers (µmol/l)**					
Vitamin C	n= 702	-3 (-6, 0.1)	0.06	4 (0.09, 8)	0.04
Total caro [(f)]	n= 506	-0.2 (-0.4, -0.09)	0.003	-0.05 (-0.2, 0.1)	0.6
α-carotene	n= 702	-0.006 (-0.01,0.007)	0.3	-0.002 (-0.01 ,0.01)	0.7
β-carotene	n= 748	-0.05 (-0.1, -0.008)	0.02	0.01 (-0.04, 0.07)	0.6
Lycopene	n= 746	-0.09 (-0.1, -0.03)	0.003	-0.05 (-0.1 0.02)	0.2

39 participants reported being vegetarian and were excluded from this analyses.

Table 5. The association between sleep duration categories and FV intakes, nutrients and associated biomarkers for adults from the NDNS years 1-4 after further adjusting for BMI and physical activity.

		**Sleep categories compared to the reference group (7-8hours/day)**			
**Model 2 (n=1171)**		**Short sleepers <7 hours/day**		**Long sleepers >8 hours/day**	
**FV intake**		**Mean difference (95%CI)**	**P value**	**Mean difference (95%CI)**	**P value**
Total fruit[(a)] (g/day)		-17(-29,-5)	0.006	-19 (-34, -3)	0.01
Total veg[(b)] (g/day)		-3 (-16, 9)	0.5	-10 (-26, 6)	0.2
FV portions[(c)]		-0.2 (-0.5, -0.03)	0.09	-0.3 (-0.6, -0.03)	0.08
5-a-day portions[(d)]		-0.2 (-0.5, -0.03)	0.08	-0.3 (-0.5 ,0.05)	0.09
Total FV [(e)](g/day)		-21 (-41, -1)	0.03	-29 (-54, -4)	0.02
**Nutrients (mg)**					
Vitamin C diet only		-1 (-8, 5)	0.7	-5 (-14, 3)	0.2
Vitamin C *		-9 (-26, 8)	0.3	-12 (-34, 8)	0.2
**Biomarkers (μmol/l)**					
Vitamin C	n= 550	-2 (-6, 0.7)	0.1	4 (-0.01, 9)	0.05
Total caro [(f)]	n= 440	-0.2 (-0.4, -0.05)	0.01	-0.04 (-0.2 , 0.2)	0.7
α-carotene	n= 563	-0.002 (-0.01, 0.01)	0.7	-0.002 (-0.02, 0.01)	0.7
β-carotene	n= 588	-0.03 (-0.09 ,0.01)	0.1	0.03 (-0.04, 0.1)	0.4
Lycopene	n= 585	-0.09(-0.1, -0.02)	0.007	-0.01(-0.1, 0.07)	0.7

Physical activity was time spent at moderate or vigorous physical activity (hour/day).

Table 6. The association between weekday/weekend sleep duration categories and FV intakes and associated biomarkers for adults from the NDNS year 1-4.

**Weekday sleep categories compared to reference group (7-8 hours/day)**				
**Model 2 (n=1610)**	**Short sleepers <7 hours/day**		**Long sleepers >8 hours/day**	
**FV intake**	**Mean difference (95%CI)**	**P value**	**Mean difference (95%CI)**	**P value**
Total fruit [(a)] (g/day)	-13 (-23, -2)	0.01	-28 (-44, -12)	0.001
Total veg [(b)] (g/day)	-8 (-19, 2)	0.1	-16 (-32, -0.1)	0.04
FV portions[(c)]	-0.2 (-0.4,-0.02)	0.03	-0.5 (-0.8, -0.1)	0.003
5-a-day portions[(d)]	-0.2 (-0.4 , -0.03)	0.02	-0.5 (-0.8, -0.1)	0.004
Total FV[(e)] (g/day)	-21 (-38, -4)	0.01	-44 (-70, -19)	0.001
**Nutrients (mg)**				
Vitamin C diet only	-3 (-10, 2)	0.2	-8 (-18, 0.9)	0.07
Vitamin C **	-4 (-18, 9)	0.5	-18 (-38, 2)	0.08
**Biomarkers**(µmol/l)				
Vitamin C n=718	-4 (-7, -0.9)	0.01	0.04 (-4, 4)	0.9
Total caro[(f)] n= 520	-0.2 (-0.4, -0.06)	0.008	-0.09 (-0.3, 0.1)	0.4
α-carotene n=719	-0.005 (-0.01, 0.007)	0.3	-0.004 (-0.02, 0.01)	0.6
β-carotene n= 765	-0.05 (-0.1, -0.01)	0.01	0.01 (-0.06,0.08)	0.7
Lycopene n= 763	-0.08 (-0.1, -0.01)	0.01	-0.08 (-0.1,0.01)	0.09
**Weekend sleep categories compared to reference group (7-8 hours/day)**				
**Model 2 (n=1610)**	**Short sleepers <7 hours/day**		**Long sleepers >8 hours/day**	
**FV intake**	**Mean difference (95%CI)**	**P value**	**Mean difference (95%CI)**	**P value**
Total fruit [(a)] (g/day)	-10 (-22, 1)	0.07	-3 (-15, 8)	0.5
Total veg [(b)] (g/day)	-14 (-26, -2)	0.01	-6 (-18, 6)	0.3
FV portions[(c)]	-0.3 (-0.5 ,-0.05)	0.01	-0.1 (-0.3, 0.1)	0.4
5-a-day portions[(d)]	-0.2 (-0.5,-0.03)	0.02	-0.04 (-0.3, 0.2)	0.7
Total FV[(e)] (g/day)	-24 (-43, -6)	0.010	-9 (-29, 9)	0.3
**Nutrients (mg)**				
Vitamin C diet only	-4 (-11, 2)	0.2	4 (-2, 11)	0.2
Vitamin C *	-8 (-23, 6)	0.2	-1 (-17, 13)	0.8
**Biomarkers**(µmol/l)				
Vitamin C n=717	-4 (-7, -0.6)	0.020	1 (-2, 5)	0.3
Total caro[(f)] n= 519	-0.2 (-0.4, -0.1)	0.003	-0.05 (-0.2, 0.1)	0.6
α-carotene n= 718	-0.004 (-0.01, 0.009)	0.5	-0.001 (-0.01, 0.01)	0.8
β-carotene n= 764	-0.05(-0.1, -0.0006)	0.04	0.01 (-0.04,0.07)	0.6
Lycopene n= 762	-0.09 (-0.1, -0.03)	0.003	-0.05 (-0.1,0.01)	0.1

Table 7. The association between sleep duration categories and FV intakes, nutrients and associated biomarkers for adults from the NDNS years 1-4 stratified by BMI.

Short sleepers (<7h/d) and Long sleepers (>8h/d) compared to the reference group (7-8 h/d) stratified by BMI												
**BMI categories**	**BMI 18.5-25 n= 524**				**BMI 25-30 n= 525**				**BMI ≥30 n= 438**			
	**Short sleepers <7 h/d**		**Long sleepers >8 h/d**		**Short sleepers <7 h/d**		**Long sleepers >8 h/d**		**Short sleepers <7 h/d**		**Long sleepers >8 h/d**	
**FV intake Model 2**	**Mean difference (95%CI)**	**P value**	**Mean difference (95%CI)**	**P value**	**Mean difference (95%CI)**	**P value**	**Mean difference (95%CI)**	**P value**	**Mean difference (95%CI)**	**P value**	**Mean difference (95%CI)**	**P Value**
Total fruit[a] (g/day)	-22 (-45,-0.4)	0.04	-26 (-50,-1)	0.03	-8 (-26,9)	0.3	-4 (-27,18)	0.7	-16(-36,2)	0.09	-22 (-51,5)	0.1
Total veg[b] (g/day)	-23 (-45,-1)	0.04	-11 (-35,12)	0.3	3 (-15,23)	0.6	-3 (-27,21)	0.8	-21(-41,-1)	0.03	-24(-53, 3)	0.09
FV portions[c]	-0.6 (-1,0.2)	0.004	-0.4 (-0.8,0.08)	0.1	-0.01 (-0.3,0.3)	0.9	-0.1 (-0.6,0.3)	0.6	-0.3(0.7,0.03)	0.07	-0.4 (-1,0.1)	0.1
5-a-day portions[d]	-0.6 (-1,-0.1)	0.006	-0.3 (-0.8,0.1)	0.1	-0.02 (-0.4,0.3)	0.9	-0.09 (-0.6,0.4)	0.6	-0.3(-0.8,0.04)	0.07	-0.3(-0.9, 0.2)	0.2
Total FV[e](g/day)	-46 (-81,-11)	0.009	-37 (-75,0.1)	0.05	-4 (-33,24)	0.7	-7 (-45,30)	0.6	-38 (-69,-7)	0.01	-47(-92,-2)	0.03
**Nutrients (mg/d)**												
Vitamin C diet only	-6 (-18,5)	0.2	-2 (-15,10)	0.7	-5 (-17,6)	0.3	2 (-13,17)	0.7	-5 (-18,7)	0.4	-4 (-23,14)	0.6
Vitamin C *	4 (-21,31)	0.7	3 (-24,32)	0.7	-26 (-53,0.1)	0.04	-29 (-63,5)	0.1	-0.1(-23,23)	0.9	-0.4(-34,33)	0.9
**Biomarkers (µmol/l)**												
Vitamin C	-3(-9,2)	0.2	2 (-3,9)	0.4	0.06 (-6,5)	0.9	5(-2,12)	0.2	-4(-9,1)	0.1	2(-5,10)	0.5
Total carot[f]	-0.4 (-0.7,-0.1)	0.007	-0.2 (-0.5,0.1)	0.2	-0.2 (-0.4,-0.006)	0.04	-0.1 (-0.4,0.1)	0.2	-0.1(-0.3,0.09)	0.2	-0.08(-0.4,0.2)	0.5
α-carotene	-0.01 (-0.04,0.009)	0.2	-0.01 (-0.04,0.01)	0.4	-0.007 (-0.02, 0.01)	0.4	-0.004 (-0.03,0.02)	0.6	0.001(-0.01,0.02)	0.8	-0.01(-0.04,0.01)	0.3
β-carotene	-0.07 (-0.1, -0.03)	0.1	-0.01 (-0.1, 0.1)	0.7	-0.03 (-0.1,0.03)	0.3	0.04(-0.05,0.1)	0.3	-0.002(-0.06,0.05)	0.9	-0.04(-0.1,0.04)	0.3
Lycopene	-0.1 (-0.2,0.08)	0.2	-0.02(-0.2,0.1)	0.8	-0.02 (-0.1, 0.1)	0.7	-0.03 (-0.2, 0.1)	0.7	0.03(-0.1,0.2)	0.6	0.1(-0.1,0.3)	0.4

Longstanding illness description

1. Cancer (neoplasm) including lumps, masses, tumours, and growths and benign (non-malignant) lumps and cysts.
2. Diabetes including hyperglycaemia.
3. Other endocrine/ metabolic.
4. Mental illness/anxiety/depression/nerves.
5. Mental handicap.
6. Epilepsy/fits/convulsions.
7. Migraine/ headaches.
8. Other problems of nervous system.
9. Cataract/ poor eye sight/ blindness.
10. Other eye complaints.
11. Poor hearing/deafness.
12. Tinnitus/noises in the ear.
13. Meniere’s disease/ear complaints causing balance problems.
14. Other ear complaints.
15. Stroke/cerebral haemorrhage/cerebral thrombosis.
16. Heart attack/angina.
17. Hypertension/high blood pressure/blood pressure.
18. Other heart problems.
19. Piles/haemorrhoids including Varicose Veins in anus.
20. Varicose veins/phlebitis in lower extremities.
21. Other blood vessels/embolic.
22. Bronchitis/emphysema.
23. Asthma.
24. Hay fever.
25. Other respiratory complaints.
26. Stomach ulcer/ulcer/abdominal hernia/rupture.
27. Other digestive complaints (stomach, liver, pancreas, bile ducts, small intestine).
28. Complaints of bowel/colon (large intestine, caecum, bowel, colon, rectum).
29. Complaints of teeth/mouth/tongue.
30. Kidney complaints.
31. Urinary tract infection.
32. Other bladder problems/incontinence.
33. Reproductive system disorders.
34. Arthritis/rheumatism/fibrosis.
35. Back problems/slipped disc/spine/neck.
36. Other problems of bones/joints/muscles.
37. Infectious and parasitic disease.
38. Disorders of blood and blood forming organs and immunity disorders.
39. Skin complaints.
40. Other complaints.
41. Unclassifiable (no other codable complaint).
42. Complaint no longer present.

Table 1-7 legends

Model 2 adjusted for age, gender, socio-economic status, smoking, ethnicity and food energy. G, gram, CI, Confidence interval, veg, vegetables, mg, milligram, µmol, micromole, l, litre, n, number, FV, fruits and vegetables, BMI, body mass index

a) Total fruit (not including juice) = Fruit(g)+Dried fruit (g)+ Smoothie fruit (g)
b) Total vegetables= Beans (g) + Brassicaceae (g) + Other Vegg + Tomatoes (g) + Tomato Puree (g) +Yellow Red Green (g).
c) FV portions= (Fruit (g) + Driedfruitx3_mean + Tompureex5 mean + beans max mean+ Brassicaceae (g) + Yellow Red Green (g) + Other veg (g) + Tomatoes (g)) / 80 .
d) 5-a-day portions(portions/day)= Fruit veg portions + Fruit juice portions+ Smoothie Fruit portions
e) Total FV (not including juice) = Total fruit +Total vegetables
f) Total carotenoids = Lutein + alpha-cryptoxanthin + beta-cryptoxanthin+ lycopene + alpha-carotene + beta-carotene
   *Vitamin C including supplements
