# Supplementary material for: The relationship between sleep duration and fruit/vegetable intakes in UK adults: a cross-sectional study from the National Diet and Nutrition Survey
Source: BMJ Open. 2018 Apr 27;8(4):e020810. doi: 10.1136/bmjopen-2017-020810 (PMC5922510; doi:10.1136/bmjopen-2017-020810)
Supplement: Author's manuscript [file bmjopen-2017-020810.draft_revisions.pdf]

BMJ Open

BMJ Open is committed to open peer review. As part of this commitment we make the peer review history of every article we publish publicly available.

When an article is published we post the peer reviewers' comments and the authors' responses online. We also post the versions of the paper that were used during peer review. These are the versions that the peer review comments apply to.

The versions of the paper that follow are the versions that were submitted during the peer review process. They are not the versions of record or the final published versions. They should not be cited or distributed as the published version of this manuscript.

BMJ Open is an open access journal and the full, final, typeset and author-corrected version of record of the manuscript is available on our site with no access controls, subscription charges or pay-per-view fees (http://bmjopen.bmj.com).

If you have any questions on BMJ Open's open peer review process please email info.bmjopen@bmj.com

BMJ Open

The relationship between sleep duration and fruit/vegetable intake in UK adults: a cross-sectional study from The National Diet and Nutrition Survey.

Journal:	*BMJ Open*
Manuscript ID	bmjopen-2017-020810
Article Type:	Research
Date Submitted by the Author:	24-Nov-2017
Complete List of Authors:	Noorwali, Essra; University of Leeds Faculty of Biological Sciences,<br>Cade, Janet; University of Leeds, School of Food Science<br>Burley, Victoria; University of Leeds, School of Food Science and Nutrition<br>Hardie, Laura; University of Leeds, Leeds Institute of Cardiovascular and Metabolic Medicine, School of Medicine
Keywords:	EPIDEMIOLOGY, Nutrition < TROPICAL MEDICINE, PUBLIC HEALTH

SCHOLARONE™
Manuscripts

For peer review only

Title Page

The relationship between sleep duration and fruit/vegetable intake in UK adults: a cross-sectional study from The National Diet and Nutrition Survey.

Essra A. Noorwali [(1) (2)], Janet E. Cade [(1)], Victoria J. Burley [(1)], Laura J. Hardie[(3)]

[(1)] Nutritional Epidemiology Group, School of Food Science and Nutrition, University of Leeds, Leeds LS2 9JT, UK, [(2)] Department of Clinical Nutrition, Faculty of Applied Medical Sciences, Umm Al-Qura University, P.O. Box 715, Makkah 21955, Saudi Arabia, [(3)] Division of Epidemiology and Biostatistics, Leeds Institute of Cardiovascular and Metabolic Medicine, School of Medicine, University of Leeds, Leeds, LS2 9JT, UK

Correspondence author: Essra A. Noorwali, fsean@leeds.ac.uk or eanoorwali@uqu.edu.sa

Word count: 3,000

Key words: sleep duration, fruits and vegetables, nutritional epidemiology

For peer review only - http://bmjopen.bmj.com/site/about/guidelines.xhtml

WHAT IS ALREADY KNOWN ON THIS SUBJECT?
To the best of our knowledge, only two studies have investigated the association between sleep duration and fruit/vegetable consumption among adults[1 ,2]. However, these studies used limited dietary assessment measures of fruit/vegetable consumption with intake assessed as daily servings over the past month/year. This study was needed to clarify the relationship between sleep duration and fruit/vegetable intake using detailed, valid dietary data and biomarkers among UK adults.
**WHAT THIS STUDY ADDS?**
This study is unique because it describes the non-linear association between sleep duration and fruit/vegetable intake and biomarkers using restricted cubic spline modelling using data from the National Diet and Nutrition Survey representing UK adults. Participants sleeping 7-8hours/day had the highest intakes of fruit/vegetable and the highest levels of associated biomarkers compared to short and long sleepers. This study adds to the growing body of evidence linking sleep to a healthy lifestyle.

For peer review only

Abstract

Objectives: There is increasing evidence to suggest an association between sleep and diet. The aim of the present study was to examine the association between sleep duration and fruit/vegetable (FV) intakes and associated biomarkers in UK adults.

Design: cross-sectional.

Setting: data from The National Diet and Nutrition Survey.

Participants: 1612 adults aged 19-65 years. Pregnant/breastfeeding women were excluded.

Outcome measures: Sleep duration was assessed by self-report and diet was assessed by 4-day food diaries, disaggregation of foods containing FV into their components was conducted to determine total FV intakes. Sleep duration was divided to: short (<7 h/d), reference (7-8 h/d) and long (>8 h/d) sleep periods. Multiple regression adjusting for confounders was used for analyses where sleep duration was the exposure and FV intakes and their associated biomarkers were the outcomes.

Results: In adjusted models , Long Sleepers (LS) consumed on average 28 (95%CI -50,-6, p=0.01) g/d less of total FV compared to Reference Sleepers (RS), whereas Short Sleepers (SS) consumed 24 g/d less (95%CI -42,-6, p=0.006) and had lower levels of FV biomarkers (total carotenoids, β-carotene and lycopene) compared to RS. The association between sleep duration and FV intake was non-linear (p<0.001) with RS having the highest intakes. The associations between sleep duration and plasma total carotenoids (p=0.0035), plasma vitamin C (p=0.009) and lycopene (p<0.001) were non-linear with RS having the highest levels.

Conclusions: These findings show a link between sleep duration and FV consumption. This may have important implications for lifestyle and behaviour change policy.

Key words: Sleep, fruits and vegetables, nutritional epidemiology

For peer review only

Strengths and limitations of this study

- The four-day estimated diary has been validated against several biomarkers and demonstrated better estimates of average intakes compared to other dietary assessment methods.
- The disaggregation of foods containing FV into their components helped in assessing total FV intake.
- The self-report of sleep duration was based on memory and could cause over-reporting.
- Cross-sectional studies do not detect causal relationships but only represent associations.

For peer review only - http://bmjopen.bmj.com/site/about/guidelines.xhtml

INTRODUCTION

The consumption of fruits and vegetables has shown to improve the overall health [3] and reduce the risk of chronic diseases[4-6] when 400 grams or more are consumed as recommended by The World Health Organization[7]. Hence, identifying lifestyle factors associated with higher intakes of FV is a public health priority.

The relationship between sleep duration and risk of obesity was reported in a recent meta-analysis with short sleep duration associated with a 45% increased risk of obesity due to several behavioural mechanisms including the reduced intake of FV[8]. Thus, it is essential to study FV consumption in relation to sleep duration.

There are limited studies assessing the association between sleep duration and FV consumption in UK adults using validated and detailed dietary data [9]. To our knowledge, this study is the first to use data that disaggregated foods containing FV into their components which helped in assessing total FV intake. Therefore, this study aims to assess the relationship between sleep duration and daily FV consumption and their associated biomarkers in adults aged 19-65 years using data of the National Diet and Nutrition Survey (years 1-4) that represents the UK population.

METHODS

Study population

The National Diet and Nutrition Survey (NDNS) is a government-commissioned rolling programme that started in 1992 to assess the diet, nutrient intake and nutritional status of the UK population[10]. This study used combined data from years 1, 2, 3 and 4 of the rolling programme (2008/09 – 2011/12) for adults aged 19-65 years old [11]. Between April 2008 and March 2011, random samples of 21,573 addresses from 799 postcode sectors were drawn from the UK Postcode Address File. Households were selected randomly and within the household either one adult (aged 19 years and over) and one child (aged 1.5 to 18 years), or one child were randomly selected to participate[10].

Dietary records

The NDNS survey assessed dietary intake using a four-day estimated diary that included instructions on how to complete the diary, as described in detail elsewhere[10]. Participants were asked to record food and drink consumed both at home and away from home for four consecutive days. Participants were asked to record portion sizes as instructed or in

For peer review only - http://bmjopen.bmj.com/site/about/guidelines.xhtml

household measures. They were asked to record brand names, ingredients and quantities, cooking methods, leftovers and dietary supplements. Dietary intake was calculated by trained coders and editors in the Diet In Nutrients Out dietary assessment system which calculates food and beverage nutrient intake based on data for >6,000 foods[12]. Detailed information on data coding is provided elsewhere [10].

Fruit and vegetable intake

To determine the total intakes of FV, disaggregation of foods containing FV into their components was conducted by NDNS. FV content of soft drinks, confectionery, cakes (including fruit cake) and biscuits, sugar preserves (including jam) and sweet spreads, savoury snacks and ice cream were excluded from the estimates because they fell into the "high fat/high sugars" segment of the "eat well plate" [13]. The disaggregation process and the calculation of "5-a-day" portions using disaggregated data is described elsewhere [10 ,14].

Blood Sampling (Fruit and vegetable biomarkers)

Samples were collected between February 2008 and July 2012; Years 1 to 4 of the NDNS Rolling Programme. In Year 1 there was a two week time lag between the start of the interviewer and nurse stages. From Year 2 onwards, the gap was extended, to an average of eight weeks, with the aim of increasing nurse stage response rates. Participants were asked a series of screening questions prior to venepuncture to assess their eligibility for giving a blood sample. Participants with a bleeding or clotting disorder or those taking anti-coagulant medications were excluded from providing a blood sample. The blood taking procedures including collection, processing, analysing and quality control of the blood samples is explained in further detail elsewhere[10]. This study considered available biomarker measurements related to FV consumption namely plasma vitamin C, total carotenoids, α-carotene, β-carotene and lycopene. The detailed procedure for vitamin C and carotenoid analysis is described elsewhere[10].

Sleep Duration

Participants were asked about sleep duration in the following form for week nights and weekends by using a computer assisted personal interview program:

"Over the last seven days, that is since (date) how long did you usually sleep for on weeknights, that is, Sunday to Thursday nights?"

For peer review only - http://bmjopen.bmj.com/site/about/guidelines.xhtml

"And over the last seven days, how long did you usually sleep for on a weekend that is Friday and Saturday nights?"

An average time per night was sought and if respondents worked on night shifts during the last two weeks/weekends, the average time slept during the day should be entered. For this study, two separate variables were generated for sleep duration based on weekdays and weekends for all adults aged 19-65 from year 1-4 in NDNS. Average sleep duration for weekdays and weekends was calculated using the following equation ((minutes slept during the week* 5) + (minutes slept during weekends *2))/7. Sleep duration was categorised based on the literature [15-17] to SS (<7 hours (420 minutes)), RS (7-8 hours (≥ 420 minutes and ≤ 480 minutes)) and LS (> 8 hours (>480 minutes)).

Statistical analysis

Descriptive statistics such as means and proportions were conducted to describe adults from the NDNS according to sleep duration categories. P values of < 0.05 represent statistical significance. Multiple regression analysis was used to assess the relationship between sleep duration, FV intake and biomarkers. Model 1 included adjustment for age and gender only whereas model 2 was adjusted for potential confounders that were identified after the development of a directed acyclic graph these were age, gender, socio-economic status (SES) assessed by National Statistics Socio-economic Classification including 8 categories[18], smoking status [19-22] (current, ex-smoker and never), ethnicity (white, non-white) and energy intake from food. In all analyses, sleep duration was used as the exposure and FV intakes and biomarkers were the outcomes.

We used restricted cubic splines to model non-liner relationships between sleep duration as a continuous exposure (h/day) and total FV intakes as the outcomes (g/d). The splines comprised 4 polynomial segments separated by 5 knots (at the following percentiles of sleep duration 5, 27.5, 50, 72.5 and 95 as recommended by Harrell[23]) with linear regions before the first knot and after the last. The splines for biomarkers comprised 2 polynomial segments separated by 3 knots due to the small number of samples (at the following percentiles of sleep duration 10, 50 and 90 as recommended by Harrell [23]).

Sensitivity analyses were conducted including 1) considering weekdays and weekends separately; and separate analyses were conducted after 2) excluding participants who consumed vitamins, minerals or/and supplements in the previous year (526 participants); 3)excluding those who self-reported currently having a longstanding illness (547

For peer review only - http://bmjopen.bmj.com/site/about/guidelines.xhtml

participants); 4) excluding those taking prescribed medicines (566 participants) 5)excluding those who reported being vegetarian (39 participants) 6) including body mass index (BMI) as an additional adjustment to the potential confounders in model 2. Statistical analyses were conducted using IC Stata 12 / 13 statistical software, missing data were automatically dropped.

RESULTS

General characteristics of NDNS adult participants aged 19-65 years according to sleep duration category are shown in Table 1. 80 participants were excluded from the analyses due to lack of sleep data or pregnancy/breastfeeding (Fig 1). The 1612 adults included in the study had a mean age of 43 years (95%CI 43, 44) and a mean BMI of 25 (95%CI 25, 26). 33% (n=539) of the participants were SS, 49 % of the participants (n= 788) were RS and 18% (n=285) of the participants were LS. In total, 57% (95%CI 55, 60) of the participants were female, 90% (95%CI 89, 92) were white, 46% (95%CI 43, 49) reported taking prescribed medicines and 54% (95%CI 52, 57) never smoked.

Concerning FV consumption, 35% (95%CI 31, 38) of RS consumed 5 or more portions/day of FV whereas 25% (95%CI 21, 31) of LS and 28% (95%CI 24, 32) of SS consumed 5 or more portions of FV/day. LS consumed a mean of 250 (95%CI 233,267) g/d of total FV, RS consumed a mean of 309 (95%CI 297,322) g/d of total FV whereas SS had a mean intake of 276 (95%CI 261, 291) g/d of total FV(Table 1).

For peer review only

For peer review only - http://bmjopen.bmj.com/site/about/guidelines.xhtml

Table 1. General characteristics of adults from the NDNS years 1-4 according to sleep duration category.

Characteristics	Sleep Categories <7 hours/day (SS)	7-8 hours/day (RS)	> 8 hours/day (LS)	Total
**Observations (n)**	539	788	285	1612
	**Mean (95%CI)**	**Mean (95%CI)**	**Mean (95%CI)**	**Mean (95%CI)**
**Age (Years)**	45 (44, 46)	44 (43, 45)	39 (38 ,40)	43 (43,44)
**BMI**	26 (25,27)	25 (25,26)	24 (23, 25)	25 (25,26)
**Food energy**	1712 (1665, 1758)	1769 (1731,1807)	1645 (1587,1703)	1727 (1701,1752)
**Equivalised household income**	33K (31K, 35K)	34K (32K, 36K)	29K (26K,32K)	33K (32K, 34K)
**Fruit (g/d)**	98 (89, 106)	115 (107, 124)	82 (73, 92)	103 (98, 108)
**Vegetables (g/d)**	178 (168, 188)	194 (187, 201)	168 (157, 180)	185 (180, 190)
**Total FV (g/d)**	276 (261, 291)	309 (297, 322)	250 (233, 267)	287 (279, 296)
**Plasma vitamin C (µmol/l)**	48 (45, 51)	53 (51, 55)	56 (53, 59)	51 (50, 53)
**Plasma total carotenoids (µmol/l)**	2.2 (2.1, 2.4)	2.5 (2.4, 2.7)	2.4 (2.2 ,2.7)	2.4 (2.3, 2.5)
**Plasma Lycopene (µmol/l)**	0.62 (0.57, 0.67)	0.73 (0.69 , 0.77)	0.69 (0.61,0.76)	0.69 (0.66 , 0.72)
	**% (95%CI)**	**% (95%CI)**	**% (95%CI)**	**% (95%CI)**
**Sex (Female)**	55 (51, 59)	56 (52, 59)	64 (58, 69)	57 (55, 60)
**Ethnicity (White)**	92 (90, 94)	89 (86, 91)	88 (84, 91)	90 (89, 92)
**Has longstanding illness (Yes)**	37 (33, 41)	30 (26, 32)	39 (33, 45)	34 (32, 36)
**Taking prescribed medicine (Yes)**	47 (42, 51)	43 (38, 46)	53 (46, 60)	46 (43,49)
**Employer (Full or part-time employment)**	68 (64, 72)	72 (68, 75)	62 (56, 67)	69 (67, 71)
**SES (Lower managerial and professional)**	28 (24,32)	28 (25,31)	22 (17,27)	27 (24,29)
**Smoking (Never)**	51 (46,55)	57 (53, 60)	54 (48, 60)	54 (52,57)
**Consuming 5 or more portions of FV/day (Yes)**	28 (24, 32)	35 (31, 38)	25 (21, 31)	31 (29, 33)
**Vegetarian (Yes)**	2 (1,3)	3 (2,4)	0.7 (0.1, 2)	2 (1,3)
**Has one child aged between 0-4 years**	15 (12, 18)	13 (10,15)	12 (8,16)	13 (12,15)
**Frequency of drinking alcohol in past 12 months (once or twice a week or month)**	45 (40, 49)	48 (44, 51)	50 (44, 55)	47 (45, 50)

n, number CI, Confidence interval, BMI, Body mass index, SS, short sleepers, RS, reference sleepers, LS, long sleepers, g, gram, d, day, µmol, micromole, l, litre, FV, fruits and vegetables.

For peer review only - http://bmjopen.bmj.com/site/about/guidelines.xhtml

In adjusted analyses (model 2), SS and LS ate less fruit (g/d), FV portions and total FV (g/d) compared to RS (Table 2). SS ate on average 13 g/d (95%CI -24, -2, p=0.01) less total fruit, 0.2 (95%CI -0.5, -0.06, p=0.01) less portions/d of FV and 24 g/d (95%CI -42,-6, p=0.006) less total FV. LS consumed on average 16 g/d (95%CI -30, -2, p=0.01) less total fruit, 0.2 (95%CI -0.5, 0.01, p= 0.06) less portions/d of FV and 28 g/d (95%CI -50,-6, p=0.01) less total FV. In model 1 SS had on average 17 g/d (95%CI -29,-5, p=0.004), LS 19 g/d (95%CI -34, -4, p=0.009) less vegetable intake compared to RS but the differences became borderline significant with further adjustment.

In model 2, no significant difference between groups for vitamin C as a nutrient and borderline significant effect in circulating levels of vitamin C. However, SS had 0.2 μmol/l lower plasma total carotenoids (95%CI -0.4, -0.08, p=0.004), 0.05 μmol/l lower plasma β-carotene (95%CI -0.1, -0.009, p=0.01) and 0.08 μmol/l lower plasma lycopene (95%CI -0.1,-0.02, p=0.005) compared to RS. This was confirmed with SS having less intake of tomatoes compared to RS in adjusted models (-5g/d, 95%CI -9, -0.1, p=0.04). SS had a mean intake of 42 g/d (95%CI 38, 46) of tomatoes, RS had 48 g/d (95%CI 45, 51) and LS had 41 g/d (95%CI 36, 46).

For peer review only

For peer review only - http://bmjopen.bmj.com/site/about/guidelines.xhtml

Table 2. The association between sleep duration categories, FV intakes and their biomarkers of adults from the NDNS years 1-4.

		**Short sleepers (<7h/d) and Long sleepers (>8h/d) compared to the reference group (7-8 h/d)**											
**Models**		**Model 1 (n=1612)**						**Model 2 (n= 1610)**					
		**Short sleepers <7 hours/day**			**Long sleepers >8 hours/day**			**Short sleepers <7 hours/day**			**Long sleepers >8 hours/day**		
**FV intake**		**Difference of mean**	**95%CI**	**P value**	**Difference of mean**	**95%CI**	**P value**	**Difference of mean**	**95%CI**	**P value**	**Difference of mean**	**95%CI**	**P value**
Total fruit[(a)] (g/day)		-19	-31, -8	0.001	-24	-38 ,-10	0.001	-13	-24, -2	0.01	-16	-30, -2	0.01
Total veg[(b)] (g/day)		-17	-29 , -5	0.004	-19	-34 ,-4	0.009	-10	-21, 0.5	0.06	-11	-25, 2	0.09
FV portions[(c)]		-0.4	-0.6, -0.2	<0.001	-0.5	-0.8, -0.1	0.001	-0.2	-0.5, -0.05	0.01	-0.2	-0.5 ,-0.01	0.04
5-a-day portions[(d)]		-0.4	-0.7, -0.2	<0.001	-0.5	-0.8, -0.1	0.002	-0.2	-0.5, -0.06	0.01	-0.2	-0.5, 0.01	0.06
Total FV[(e)](g/day)		-37	-56, -18	<0.001	-44	-67, -20	<0.001	-24	-41, -6	0.006	-28	-50, -6	0.01
**Nutrients (mg/d)**													
Vitamin C diet only		-9	-16 , -2	0.01	-10	-19, -1	0.02	-5	-12, 1	0.1	-4	-12, 4	0.3
Vitamin C *		-13	-27, 0.8	0.06	-21	-39, -3	0.01	-7	-21, 6	0.2	-12	-30, 5	0.1
**Biomarkers (µmol/l)**													
Vitamin C	n= 717	-4	-8, -1	0.006	3	-1 , 7	0.1	-2	-6, 0.3	0.07	4	0.1, 8	0.04
Total carot[(f)]	n= 519	-0.2	-0.4, -0.1	0.002	-0.1	-0.3, 0.1	0.3	-0.2	-0.4, -0.08	0.004	-0.06	-0.2 ,0.1	0.5
α-carotene	n= 718	-0.01	-0.02,0.003	0.1	-0.009	-0.02 , 0.007	0.2	-0.006	-0.01, 0.007	0.3	-0.004	-0.02, 0.01	0.6
β-carotene	n= 764	-0.07	-0.1, -.02	0.003	-0.01	-0.07 , 0.05	0.7	-0.05	-0.1 ,-0.009	0.01	0.009	-0.05, 0.07	0.7
Lycopene	n= 762	-0.1	-0.1 ,-0.04	0.001	-0.06	-0.1 ,0.01	0.09	-0.08	-0.1, -0.02	0.005	-0.05	-0.1, 0.02	0.1

Model 1 adjusted for age and gender.
Model 2 adjusted for age, gender, socio-economic status, smoking, ethnicity and food energy.
G, gram, CI, Confidence interval, veg, vegetables, mg, milligram, µmol, micromole, l, litre, n, number, FV, fruits and vegetables

a) Total fruit (not including juice) = Fruit(g)+Dried fruit (g)+ Smoothie fruit (g)
b) Total vegetables= Beans (g) + Brassicaceae (g) + Other Vegg + Tomatoes (g) + Tomato Puree (g) +Yellow Red Green (g).
c) FV portions= (Fruit (g) + Driedfruitx3_mean + Tompureex5 mean + beans max mean+ Brassicaceae (g) + Yellow Red Green (g) + Other veg (g) + Tomatoes (g)) / 80 .
d) 5-a-day portions(portions/day)= Fruit veg portions + Fruit juice portions+ Smoothie Fruit portions
e) Total FV (not including juice) = Total fruit +Total vegetables
f) Total carotenoids = Lutein + alpha-cryptoxanthin + beta-cryptoxanthin+ lycopene + alpha-carotene + beta-carotene

*Vitamin C including supplements.

For peer review only

For peer review only - http://bmjopen.bmj.com/site/about/guidelines.xhtml

Restricted cubic spline modelling (Fig 2) showed that the association between sleep duration and total FV intake (g/d) was non-linear ($p<0.001$) with participants sleeping 7-8h/d having the highest intakes compared to SS and LS. Similarly, the association between sleep duration and plasma vitamin C ($p=0.009$) (Fig 3A), total carotenoids ($p=0.0035$) (Fig 3B) and lycopene ($p<0.001$) (Fig 3C) were non-linear with RS having the highest levels.

Sensitivity analysis

Sensitivity analysis showed broadly similar results (available as supplementary material tables 1-6). Including adjustment for BMI in the fully adjusted model did not affect the results. Results of separate analysis excluding participants who consumed minerals, vitamins and/or food supplements, being vegan/vegetarian, having a longstanding illness and consuming prescribed medicines, remained similar with SS consuming less FV in comparison to RS but no difference between LS FV intakes and RS. The association between sleep duration and biomarkers were similar with SS having lower levels compared to RS and LS having higher levels of plasma vitamin C compared to RS. Results dividing the exposure into weekday and weekend sleep duration were similar, SS on average consumed less g/d of FV and had lower levels of biomarkers on weekdays and weekends compared to RS. LS on average consumed less g/d of FV on weekdays compared to RS.

For peer review only

DISCUSSION

To our knowledge, this is the first nationally representative study to examine the association between sleep duration and FV intakes using disaggregated data among UK adults. The results of this study show SS and LS have lower intakes of FV compared to RS. Results of FV biomarkers show lower levels of all plasma biomarkers except α-carotene and vitamin C in SS compared to RS in contrast to plasma vitamin C levels in LS which were higher than RS. Similar results were noted after excluding participants who had a longstanding illness, consumed prescribed medicines and those who consumed supplements, minerals or/and vitamins in the previous year. The association between sleep duration, FV intake and biomarkers were non-linear with RS having the highest intakes and levels of biomarkers compared to SS and LS as shown in the restricted cubic spline modelling. Thus, these findings suggest that among UK adults RS have the highest intakes of FV compared to SS and LS.

These results are in line with several other cross-sectional studies[1 ,15 ,16 ,24 ,25]. Although the studies differed in sample size, ethnicity, dietary assessment methods and categorisation of sleep duration, the results showed a lower intake of FV in short/long sleepers compared to RS. In a study that examined the association between sleep duration and diet quality among women following childbirth, short sleep duration was not associated with diet quality whereas long sleep duration was associated with lower consumption of total and whole fruit[26]. Katagiri *et al.* (2014) measured the association between sleep quality and diet and noted that poor sleep quality was significantly associated with low intakes of total vegetables, green/yellow vegetables and other vegetables[27]. The study suggested that the relationship of dietary intake with sleep quality is similar to that with sleep duration. Regarding the results of FV biomarkers, our results were supported by two other studies[15 ,28]. Grandner *et al.* (2013) showed a significant lower intakes of lycopene in very short sleepers (<5hours) in [28]. Beydoun *et al.* (2014) reported that short sleep duration was associated with lower serum levels of vitamin C, total carotenoids, α-carotene and β-carotene compared to RS[15].

In contrast, a recent study examined the association between sleep duration and cardiovascular risk behaviours using data from the UK Biobank found results which were inconsistent with our own. Long sleep duration was positively associated with vegetable intake[2]. These results which contrast with our findings may be due to the different assessment methods of sleep duration and FV intake. Sleep duration assessment in the UK biobank did not consider weekday and weekend sleep duration separately as conducted in the

For peer review only - http://bmjopen.bmj.com/site/about/guidelines.xhtml

NDNS study since sleep duration may differ between those days[29]. Furthermore, self-report of sleep duration may differ by question format by reporting short sleep duration when asked a single question [30]. Sleep duration in the UK biobank study was assessed by asking one question in regard to sleep every 24h whereas our study assessed sleep duration by asking two separate questions of sleep based on weeknights and weekends. FV intakes were assessed differently in the UK Biobank[2] and the NDNS survey[14]. Patterson *et al.* (2016) assessed FV intake by considering diet intake in the previous year and asking how many pieces of fresh fruit would participants eat per day and how many heaped table-spoons of vegetables participants would eat on average per day. This method was based on the UK guidelines that a portion of vegetables is three heaped tablespoons whereas the NDNS survey assessed dietary intake using a four-day estimated diary and disaggregated foods containing FV which is considered a better estimate of average intakes compared to other dietary assessment methods[31]. Additionally, this study conducted supportive biomarker analyses. In a home-based intervention study that assessed the effects of extended bedtimes on sleep duration and food desire, desire for FV was not affected by added sleep[32]. However, the study had several limitations including a small sample size which may limit the generalizability to more diverse populations. One of the major limits of the intervention study[32] is the short duration of intervention (2 weeks) which does not measure the potential effects over a longer period. Experimental studies differ from free-living individuals therefore; it is required to consider the potential for non-representative samples taking part in experimental studies.

Several mechanisms may underlie the association between sleep duration and diet intake [9 ,33]. Emotional stress, impaired decision making and preference for energy-dense foods are some of the proposed mechanisms[34]. Hormonal changes of ghrelin and leptin that were not measured in this study may be the underlying mechanism for decreased intake of FV in SS and LS. On the other hand, sleep may be promoted by foods such as kiwifruits, tart cherries, milk and chamomile tea for their impact on tryptophan availability and the synthesis of serotonin and melatonin[35]. This provides insight to the relationship between sleep and diet being potentially bi-directional.

Strengths and limitations of the study

The main strength of this study was the disaggregation of foods containing FV into their components which helps in assessing total FV intake[14]. Furthermore, the four-day

For peer review only - http://bmjopen.bmj.com/site/about/guidelines.xhtml

estimated diary has been validated against several biomarkers and demonstrated better estimates of average intakes compared to other dietary assessment methods.

This study has several limitations including the self-report of sleep duration which was based on memory and could cause over-reporting[36]. Further limitations include lack of consideration of other sleep factors such as sleep quality[27], sleep timing [37], sleep problems and chronotype[2]. In Year 1, weekend days were oversampled and in year 2 they were under-sampled to redress that however, in the years 1 to 4 combined data there still remains a slightly higher proportion of weekend days. Eating habits vary between weekdays and weekends [38] which could lead to a bias in the reporting of FV intake. The small number of participants in the obtained biomarkers was a further limitation. The association between sleep duration and FV intake is a bi-directional relationship and the causal pathways underlying the relationship cannot be detected in cross-sectional studies[27].

PUBLIC HEALTH IMPLICATIONS

Sleep duration among UK adults has been declining recently with 70% of UK adults sleeping less than 7h/night according to the Sleep Council [39]. Additionally, the intake of FV intake is decreasing among UK adults with only 30% of them meeting the 5-a-day recommendation according to the NDNS results provided by Public Health England[13]. These trends highlight the importance of translating the scientific evidence focusing on the relationship between sleep and diet into practical messages that can help the public to prevent chronic diseases. It is required to make different populations aware of the relationship between sleep and diet by including more information in national dietary guidelines to enhance healthy lifestyle recommendations. In addition, this information can be incorporated in hospitals to educate healthcare professionals, weight-loss programs and other programs targeting improvement in overall health. This information is also essential for those caring for at risk groups such as elderly and those with chronic diseases[40].

CONCLUSIONS

The results of this study showed that sleep duration was non-linearly related to FV intakes and their biomarkers with RS having the highest intake of FV compared to SS and LS. Future interventional trials are required to incorporate objective measures of sleep to clarify the relationships between sleep and FV intakes.

For peer review only

ACKNOWLEDGMENTS

We thank the study participants and the UK Data Service.

DATA AVAILABILITY

All National Diet and Nutrition Survey Data are available online at the UK Data Service website (see https://www.gov.uk/government/collections/national-diet-and-nutrition-survey).

Ethical approval was not required for this study.

FINANCIAL SUPPORT

Essra Noorwali is in receipt of a scholarship from Umm Al-Qura University, Makkah, Saudi Arabia.

CONFLICT OF INTEREST

None.

AUTHORSHIP

The authors' contributions are as follows: Essra Noorwali was the principal investigator and contributed to the study design, data analyses, interpretation of the findings and wrote the manuscript; Victoria Burley contributed to the study design, data analyses, interpretation of findings; Laura Hardie; contributed to the study design, data analyses interpretation of findings and article revision; Janet Cade contributed to interpretation of findings and article revision. All authors read and approved the final version of the manuscript.

For peer review only

REFERENCES

1. Stamatakis KA, Brownson RC. Sleep duration and obesity-related risk factors in the rural Midwest. Prev Med 2008;**46**(5):439-44.
2. Patterson F, Malone SK, Lozano A, et al. Smoking, Screen-Based Sedentary Behavior, and Diet Associated with Habitual Sleep Duration and Chronotype: Data from the UK Biobank. Ann Behav Med 2016.
3. Wang X, Ouyang, Y., Liu, J., et al. Fruit and vegetable consumption and mortality from all causes, cardiovascular disease, and cancer: systematic review and dose-response meta-analysis of prospective cohort studies. Bmj 2014;**349**:g4490.
4. Ishihara J UM. Relationship Between Vegetables and Fruits (Antioxidant Vitamins, Minerals, and Fiber) Intake and Risk of Cardiovascular Disease. Reference Module in Biomedical Sciences 2017.
5. Liu S, Manson, J.E., Lee, I.M., et al. Fruit and vegetable intake and risk of cardiovascular disease: the Women's Health Study. The American journal of clinical nutrition 2000;**72**(4):922-28.
6. Aune D, Giovannucci E., Boffetta P., et al. Fruit and vegetable intake and the risk of cardiovascular disease, total cancer and all-cause mortality–a systematic review and dose-response meta-analysis of prospective studies. Int J Epidemiol 2016.
7. WHO Study Group on Diet Nutrition and Prevention of Noncommunicable Diseases. report of a WHO Study Group. Technical report series. Geneva, 1990:203p.
8. Wu Y, Zhai L, Zhang D. Sleep duration and obesity among adults: a meta-analysis of prospective studies. Sleep Med 2014;**15**(12):1456-62.
9. Dashti HS, Scheer FA, Jacques PF, et al. Short sleep duration and dietary intake: epidemiologic evidence, mechanisms, and health implications. Adv Nutr 2015;**6**(6):648-59.
10. Bates B, Lennox A, Prentice A, et al. National Diet and Nutrition Survey Results from Years 1, 2, 3 and 4 (combined) of the Rolling Programme (2008/2009-2011/2012) 2014:Availablefrom:https://www.gov.uk/government/uploads/system/uploads/attachment_data/file/594360/NDNS_Y1_to_4_UK_report_executive_summary_revised.pdf
11. NatCen Social Research, MRC Elsie Widdowson Laboratory, University College London (2017). Medical School. National Diet and Nutrition Survey Years 1-6, 2008/09-2013/14. [data collection]. 8th Edition.UK Data Service, [Accessed 24 April 2017]. Available from: http://doi.org/10.5255/UKDA-SN-6533-7.
12. The National Archives.The composition of foods 2002. http://tna.europarchive.org/20110116113217/http://www.food.gov.uk/science/dietarysurveys/dietsurveys/.[Accessed September 2016].
13. Eat well plate. 2013. http://www.nhs.uk/Livewell/Goodfood/Pages/eatwell-plate.aspx.[Accessed September 2016].
14. Fitt E, Mak TN, Stephen AM, et al. Disaggregating composite food codes in the UK National Diet and Nutrition Survey food composition databank. Eur J Clin Nutr 2010;**64 Suppl 3**:S32-6.

For peer review only - http://bmjopen.bmj.com/site/about/guidelines.xhtml

15. Beydoun MA, Gamaldo AA, Canas JA, et al. Serum nutritional biomarkers and their associations with sleep among US adults in recent national surveys. PLoS One 2014;**9**(8):e103490.

16. Haghighatdoost F, Karimi G, Esmaillzadeh A, et al. Sleep deprivation is associated with lower diet quality indices and higher rate of general and central obesity among young female students in Iran. Nutrition 2012;**28**(11-12):1146-50.

17. Cassidy S, Chau JY, Catt M, et al. Cross-sectional study of diet, physical activity, television viewing and sleep duration in 233,110 adults from the UK Biobank; the behavioural phenotype of cardiovascular disease and type 2 diabetes. BMJ Open 2016;**6**(3):e010038.

18. The National Statistics Socio-economic classification (NS-SEC rebased on SOC2010). http://webarchive.nationalarchives.gov.uk/20160105160709/http://www.ons.gov.uk/ons/guide-method/classifications/current-standard-classifications/soc2010/soc2010-volume-3-ns-sec--rebased-on-soc2010--user-manual/index.html.[Accessed September 2016].

19. Zhang L SJ, Caffo B, Punjabi NM. Cigarette smoking and nocturnal sleep architecture. Am J Epidemiol 2006;**15**(164(6)):529-37.

20. Jaehne A UT, Feige B, et al. How smoking affects sleep: a polysomnographical analysis. Sleep medicine 2012;**31**(13(10)):1286-92.

21. Lohse Tina SR, Matthias Bopp, et al. Heavy Smoking Is More Strongly Associated with General Unhealthy Lifestyle than Obesity and Underweight. PloS one 2016;**11**(2):e0148563.

22. Palaniappan U SL, O'Loughlin J, Gray-Donald K. . Fruit and vegetable consumption is lower and saturated fat intake is higher among Canadians reporting smoking. J Nutr 2001;**131**(7):1952-8.

23. Harrell F. *Regression Modeling Strategies : With Applications to Linear Models, Logistic Regression, and Survival Analysis* 1st ed. New York Springer, 2001.

24. Kim S, DeRoo LA, Sandler DP. Eating patterns and nutritional characteristics associated with sleep duration. Public Health Nutr 2011;**14**(5):889-95.

25. Mossavar-Rahmani Y, Jung M, Patel SR, et al. Eating behavior by sleep duration in the Hispanic Community Health Study/Study of Latinos. Appetite 2015;**95**:275-84.

26. Xiao RS, Moore Simas TA, Pagoto SL, et al. Sleep Duration and Diet Quality Among Women Within 5 Years of Childbirth in the United States: A Cross-Sectional Study. Matern Child Health J 2016.

27. Katagiri R, Asakura K, Kobayashi S, et al. Low intake of vegetables, high intake of confectionary, and unhealthy eating habits are associated with poor sleep quality among middle-aged female Japanese workers. J Occup Health 2014;**56**(5):359-68.

28. Grandner MA, Jackson N, Gerstner JR, et al. Dietary nutrients associated with short and long sleep duration. Data from a nationally representative sample. Appetite 2013;**64**:71-80.

29. Paine SJ, Gander PH. Differences in circadian phase and weekday/weekend sleep patterns in a sample of middle-aged morning types and evening types. Chronobiol Int 2016;**33**(8):1009-17.

For peer review only - http://bmjopen.bmj.com/site/about/guidelines.xhtml

30. Lauderdale DS. Survey Questions About Sleep Duration: Does Asking Separately About Weekdays and Weekends Matter? Behav Sleep Med 2014;**12**((2)):158-68.

31. Day NE, McKeown N, Wong MY, et al. Epidemiological assessment of diet: a comparison of a 7-day diary with a food frequency questionnaire using urinary markers of nitrogen, potassium and sodium. Int J Epidemiol 2001;**30**(2):309-17.

32. Tasali E, Chapotot F, Wroblewski K, et al. The effects of extended bedtimes on sleep duration and food desire in overweight young adults: a home-based intervention. Appetite 2014;**80**:220-4.

33. Lundahl A, Nelson TD. Sleep and food intake: A multisystem review of mechanisms in children and adults. J Health Psychol 2015;**20**(6):794-805.

34. Chaput JP. Sleep patterns, diet quality and energy balance. Physiol Behav 2014;**134**:86-91.

35. Peuhkuri K SN, Korpela R Diet promotes sleep duration and quality. Nutr Res 2012; **32(5)**:309-19.

36. Lauderdale DS, Knutson KL, Yan LL, et al. Self-reported and measured sleep duration: how similar are they? Epidemiology 2008;**19**(6):838-45.

37. Golley RK, Maher CA, Matricciani L, et al. Sleep duration or bedtime? Exploring the association between sleep timing behaviour, diet and BMI in children and adolescents. Int J Obes (Lond) 2013;**37**(4):546-51.

38. Decastro JM. Social Facilitation of the Spontaneous Meal Size of Humans Occurs on Both Weekdays and Weekends. Physiol Behav 1991;**49**(6):1289-91.

39. The Sleep Council.The Great British bedtime report. http://www.sleepcouncil.org.uk/wp-content/uploads/2013/02/The-Great-British-Bedtime-Report.pdf.2013.[Accessed May 2017].

40. Frank S GK, Lee-Ang Lorraine, Young Marielle C., et al. . Diet and Sleep Physiology: Public Health and Clinical Implications. Frontiers in Neurology 2017;**8**(393).

For peer review only

For peer review only - http://bmjopen.bmj.com/site/about/guidelines.xhtml

Figure 1. Participants' flow chart

Figure 2. The association between sleep duration and FV intake from the restricted cubic spline modelling. Black lines plot the predicted FV intakes with 95% confidence interval (grey shaded area) for typical participants (females, white, never smokers, lower managerial and professional occupation, using mean age (43.1) and mean food energy (1727.05)).

Figure 3. The association between sleep duration and FV biomarkers from the restricted cubic spline modelling. Black lines plot the predicted FV biomarkers values **A)** vitamin C **B)** Total carotenoids **C)** lycopene with 95% confidence interval (grey shaded area) for typical participants (females, white, never smokers, lower managerial and professional occupation, using mean age (43.1) and mean food energy (1727.05).

For peer review only

For peer review only - http://bmjopen.bmj.com/site/about/guidelines.xhtml

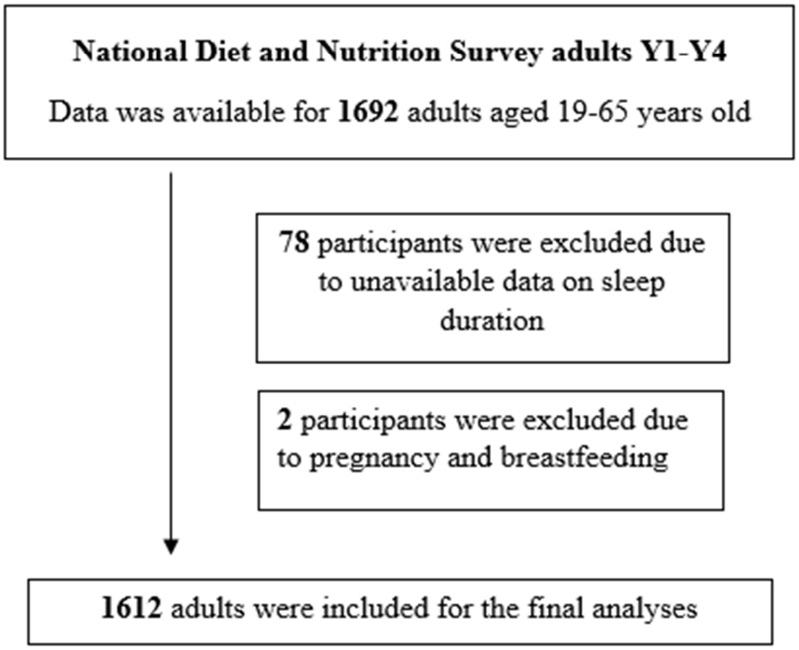

Participants' flow chart

135x98mm (96 x 96 DPI)

ew only

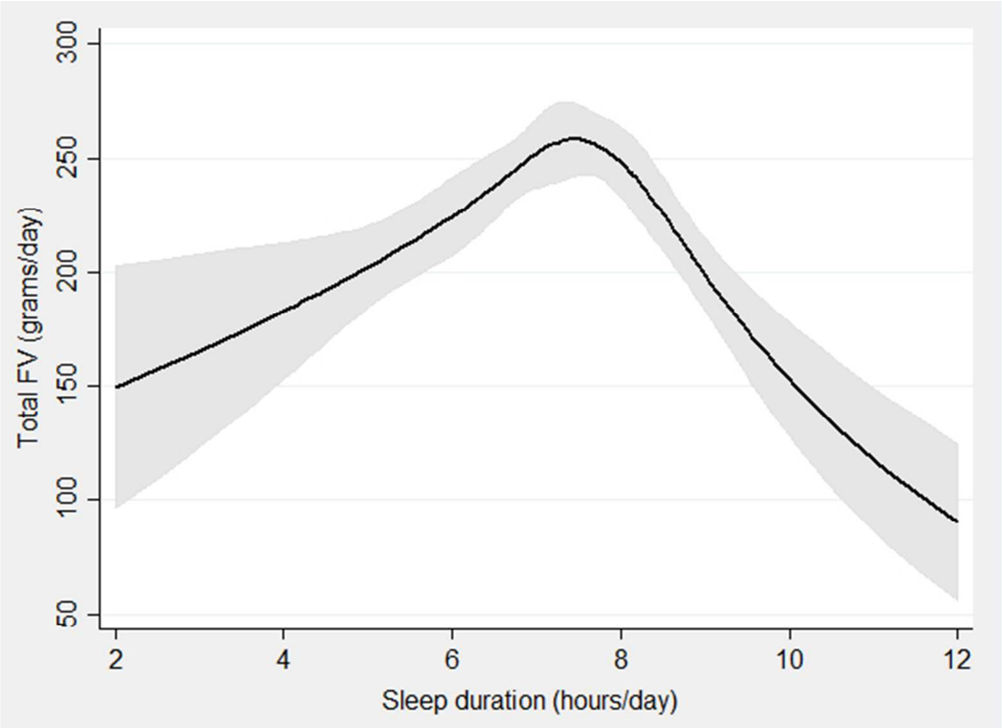

The association between sleep duration and FV intake from the restricted cubic spline modelling. Black lines plot the predicted FV intakes with 95% confidence interval (grey shaded area) for typical participants (females, white, never smokers, lower managerial and professional occupation, using mean age (43.1) and mean food energy (1727.05)).

217x158mm (72 x 72 DPI)

For peer review only - http://bmjopen.bmj.com/site/about/guidelines.xhtml

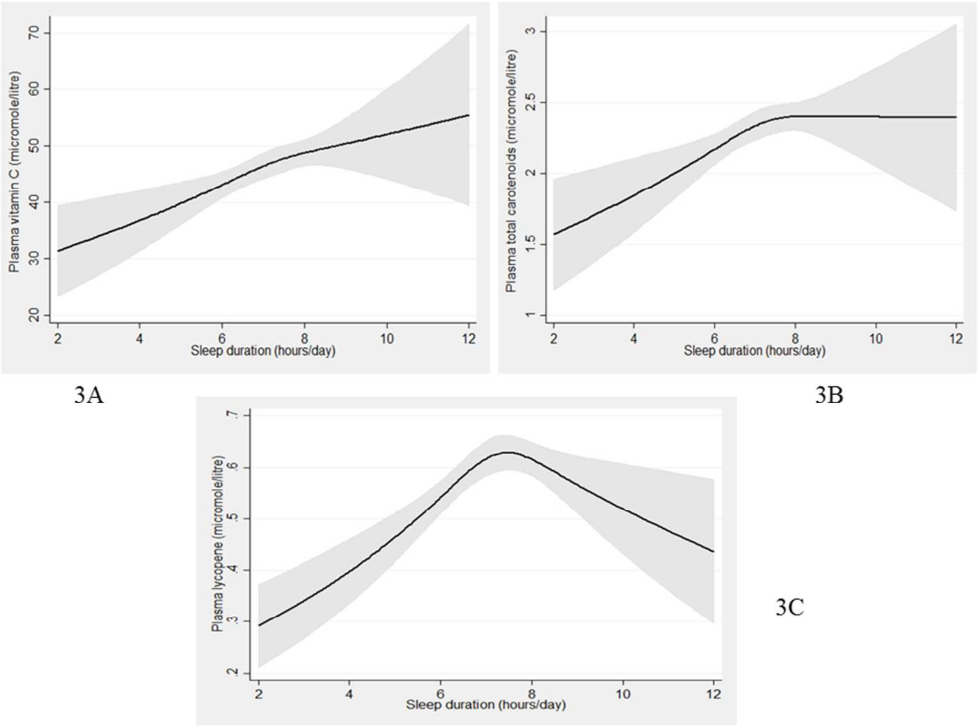

The association between sleep duration and FV biomarkers from the restricted cubic spline modelling. Black lines plot the predicted FV biomarkers values A) vitamin C B) Total carotenoids C) lycopene with 95% confidence interval (grey shaded area) for typical participants (females, white, never smokers, lower managerial and professional occupation, using mean age (43.1) and mean food energy (1727.05).

254x190mm (96 x 96 DPI)

Online supplementary material

Table 1. The association between sleep duration categories and FV intakes and associated biomarkers for adults from the NDNS years 1-4 after excluding participants who consume prescribed medicines.

		Sleep categories compared to the reference group (7-8 hours/day)					
**Model 2 (n=1044)**		**Short sleepers <7 hours/day**			**Long sleepers >8 hours/day**		
**FV intake**		**Difference of mean**	**95%CI**	**P value**	**Difference of mean**	**95%CI**	**P value**
Total fruit[(a)] (g/day)		-13	-27, -0.4	0.04	-17	-34, -0.1	0.04
Total veg[(b)] (g/day)		-10	-24, 4	0.1	-5	-24, 2	0.5
FV portions[(c)]		-0.2	-0.5, -.005	0.04	-0.1	-0.5, 0.1	0.3
5-a-day portions[(d)]		-0.2	-0.5, -0.0003	0.05	-0.1	-0.5, 0.1	0.3
Total FV [(e)](g/day)		-24	-45, -2	0.03	-23	-51, 4	0.1
**Nutrients (mg)**							
Vitamin C diet only		-5	-13, 2	0.1	-4	-15, 5.5	0.3
Vitamin C *		-7	-24, 10	0.4	-11	-33, 11	0.3
**Biomarkers (µmol/l)**							
Vitamin C	n= 375	-0.6	-5, 3	0.7	5	-0.09, 11	0.05
Total caro[(f)]	n= 294	-0.2	-0.5, -0.02	0.03	-0.08	-0.4, 0.2	0.5
α-carotene	n= 378	-0.005	-0.02,0.01	0.5	-0.009	-0.03, 0.01	0.4
β-carotene	n= 402	-0.05	-0.1, 0.02	0.1	-0.02	-0.1, 0.07	0.6
Lycopene	n= 403	-0.06	-0.1, 0.01	0.1	-0.06	-0.1, 0.04	0.2

566 participants reported taking prescribed medicines and were excluded from the analyses.

For peer review only

Table 2. The association between sleep duration categories and FV intakes and associated biomarkers for adults from the NDNS years 1-4 after excluding participants who reported consuming vitamins, minerals and/or supplements in the past year.

		Sleep categories compared to the reference group (7-8hours/day)					
**Model 2 (n=1084)**			**Short sleepers <7 hours/day**			**Long sleepers >8 hours/day**	
**FV intake**		**Difference of mean**	**95%CI**	**P value**	**Difference of mean**	**95%CI**	**P value**
Total fruit[a] (g/day)		-5	-17, 7	0.4	-10	-25. 5	0.2
Total veg[b] (g/day)		-11	-24, 1	0.09	-7	-24, 8	0.3
FV portions[c]		-0.1	-0.4, 0.07	0.1	-0.1	-0.4, 0.1	0.3
5-a-day portions[d]		-0.1	-0.4, 0.09	0.2	-0.1	-0.4, 0.2	0.4
Total FV[e](g/day)		-16	-36, 3	0.1	-17	-43, 7	0.1
**Nutrients (mg)**							
Vitamin C diet only		1	-6, 8	0.7	2	-6, 11	0.5
Vitamin C *		0.2	-8, 8	0.9	2	-7, 13	0.5
**Biomarkers (µmol/l)**							
Vitamin C	n= 462	-3	-7, 0.6	0.09	6	1, 11	0.009
Total caro [f]	n= 330	-0.2	-0.4, -0.01	0.03	0.06	-0.2 ,0.3	0.6
α-carotene	n= 461	-0.005	-0.02,0.009	0.4	0.0007	-0.01, 0.02	0.9
β-carotene	n= 492	-0.05	-0.1, 0.002	0.06	0.06	-0.01, 0.1	0.1
Lycopene	n= 490	-0.08	-0.1, -0.005	0.03	-0.04	-0.1, 0.04	0.3

526 participants reported taking vitamins, minerals or supplements in the past year and were excluded from the analyses.

For peer review only

Table 3. The association between sleep duration categories and FV intakes and associated biomarkers for adults from the NDNS years 1-4 after excluding those who have a longstanding illness.

		Sleep categories compared to the reference group (7-8hours)					
**Model 2 (n= 1063)**			**Short sleepers <7 hours**			**Long sleepers >8 hours**	
**FV intake**		**Difference of mean**	**95%CI**	**P value**	**Difference of mean**	**95%CI**	**P value**
Total fruit[(a)] (g/day)		-15	-29, -2	0.02	-9	-26, 7	0.2
Total veg[(b)] (g/day)		-14	-28, -0.04	0.04	-10	-28, 8	0.2
FV portions[(c)]		-0.4	-0.7, -0.1	0.004	-0.1	-0.5, 0.1	0.2
5-a-day portions[(d)]		-0.4	-0.7, -0.1	0.005	-.1	-0.5 , 0.1	0.3
Total FV[(e)](g/day)		-30	-52, -8	0.007	-20	-48, 8	0.1
**Nutrients (mg)**							
Vitamin C diet only		-5	-14, 2	0.1	-1	-11, 9	0.8
Vitamin C *		-12	-30, 5	0.1	-9	-32, 14	0.4
**Biomarkers (μmol/l)**							
Vitamin C	n= 474	-2	-6, 1	0.2	4	-0.4 ,9	0.07
Total caro [(f)]	n= 369	-0.3	-0.5, -0.08	0.007	-0.04	-0.3, 0.2	0.7
α-carotene	n= 477	-0.006	-0.02, 0.01	0.4	0.001	-0.01, 0.02	0.9
β-carotene	n= 506	-0.06	-0.1, -0.003	0.03	0.01	-0.06, 0.09	0.6
Lycopene	n= 505	-0.07	-0.1, 0.008	0.08	-0.05	-0.1, 0.05	0.3

547 participants reported having a longstanding illness and were excluded from the analyses.

For peer review only

Table 4. The association between sleep duration categories and FV intakes and associated biomarkers for adults from the NDNS years 1-4 after excluding those who reported being vegetarian.

		Sleep categories compared to the reference group (7-8hours/day)					
**Model 2 (n= 1571)**		**Short sleepers <7 hours/day**			**Long sleepers >8 hours/day**		
**FV intake**		**Difference of mean**	**95%CI**	**P value**	**Difference of mean**	**95%CI**	**P value**
Total fruit[(a)] (g/day)		-14	-25, -3	0.009	-9	-26, 7	0.2
Total veg[(b)] (g/day)		-11	-22, 0.1	0.05	-11	-25, 3	0.1
FV portions[(c)]		-0.3	-0.5, -0.07	0.009	-0.3	-0.5, -0.01	0.03
5-a-day portions[(d)]		-0.3	-0.5, -0.08	0.008	-0.2	-0.5, 0.008	0.05
Total FV [(e)](g/day)		-25	-43, -8	0.004	-29	-51, -7	0.009
**Nutrients (mg)**							
Vitamin C diet only		-5	-12, 0.9	0.09	-4	-12, 4	0.3
Vitamin C *		-4	-18, 8	0.4	-8	-25, 8	0.3
**Biomarkers (μmol/l)**							
Vitamin C	n= 702	-3	-6, 0.1	0.06	4	0.09, 8	0.04
Total caro [(f)]	n= 506	-0.2	-0.4 -0.09	0.003	-0.05	-0.2, 0.1	0.6
α-carotene	n= 702	-0.006	-0.01,0.007	0.3	-0.002	-0.01 , 0.01	0.7
β-carotene	n= 748	-0.05	-0.1, -0.008	0.02	0.01	-0.04, 0.07	0.6
Lycopene	n= 746	-0.09	-0.1, -0.03	0.003	-0.05	-0.1 , 0.02	0.2

39 participants reported being vegetarian and were excluded from this analyses.

For peer review only

Table 5. The association between sleep duration categories and FV intakes, nutrients and associated biomarkers for adults from the NDNS years 1-4 after further adjusting for BMI.

		**Sleep categories compared to the reference group (7-8hours/day)**					
**Model 2 (n=1610)**		**Short sleepers <7 hours/day**			**Long sleepers >8 hours/day**		
**FV intake**		**Difference of mean**	**95%CI**	**P value**	**Difference of mean**	**95%CI**	**P value**
Total fruit[(a)] (g/day)		-13	-24, -2	0.01	-16	-30, -3	0.01
Total veg[(b)] (g/day)		-10	-21, 0.7	0.06	-12	-26, 1	0.09
FV portions[(c)]		-0.2	-0.5, -0.05	0.01	-0.3	-0.5, -0.01	0.03
5-a-day portions[(d)]		-0.2	-0.5, -0.05	0.01	-0.2	-0.5 ,0.007	0.05
Total FV [(e)](g/day)		-24	-41, -6	0.007	-29	-50, -7	0.01
**Nutrients (mg)**							
Vitamin C diet only		-5	-12, 1	0.1	-4	-12, 4	0.3
Vitamin C *		-7	-21, 6	0.3	-12	-30, 5	0.1
**Biomarkers (μmol/l)**							
Vitamin C	n= 717	-2	-5, 0.6	0.1	4	0.04, 8	0.04
Total caro [(f)]	n= 519	-0.2	-0.4, -0.06	0.007	-0.06	-0.2 , 0.1	0.5
α-carotene	n= 718	-0.004	-0.01, 0.009	0.5	-0.003	-0.02, 0.01	0.6
β-carotene	n= 764	-0.04	-0.09 , -0.002	0.04	0.01	-0.05, 0.07	0.7
Lycopene	n= 762	-0.08	-0.1, -0.02	0.007	-0.05	-0.1, 0.02	0.1

For peer review only

Table 6. The association between weekday/weekend sleep duration categories and FV intakes and associated biomarkers for adults from the NDNS year 1-4.

Weekday sleep categories compared to reference group (7-8 hours/day)						
**Model 2 (n=1610)**	**Short sleepers <7 hours/day**			**Long sleepers >8 hours/day**		
**FV intake**	**Difference of mean**	**95%CI**	**P value**	**Difference of mean**	**95%CI**	**P value**
Total fruit [(a)] (g/day)	-13	-23, -2	0.01	-28	-44, -12	0.001
Total veg [(b)] (g/day)	-8	-19, 2	0.1	-16	-32, -0.1	0.04
FV portions[(c)]	-0.2	-0.4,-0.02	0.03	-0.5	-0.8, -0.1	0.003
5-a-day portions[(d)]	-0.2	-0.4 , -0.03	0.02	-0.5	-0.8, -0.1	0.004
Total FV[(e)] (g/day)	-21	-38, -4	0.01	-44	-70, -19	0.001
**Nutrients (mg)**						
Vitamin C diet only	-3	-10, 2	0.2	-8	-18, 0.9	0.07
Vitamin C **	-4	-18, 9	0.5	-18	-38, 2	0.08
**Biomarkers**(μmol/l)						
Vitamin C n=718	-4	-7, -0.9	0.01	0.04	-4, 4	0.9
Total caro[(f)] n= 520	-0.2	-0.4, -0.06	0.008	-0.09	-0.3, 0.1	0.4
α-carotene n=719	-0.005	-0.01, 0.007	0.3	-0.004	-0.02, 0.01	0.6
β-carotene n= 765	-0.05	-0.1, -0.01	0.01	0.01	-0.06, 0.08	0.7
Lycopene n= 763	-0.08	-0.1, -0.01	0.01	-0.08	-0.1, 0.01	0.09
**Weekend sleep categories compared to reference group (7-8 hours/day)**						
**Model 2 (n=1610)**	**Short sleepers <7 hours/day**			**Long sleepers >8 hours/day**		
**FV intake**	**Difference of mean**	**95%CI**	**P value**	**Difference of mean**	**95%CI**	**P value**
Total fruit [(a)] (g/day)	-10	-22, 1	0.07	-3	-15, 8	0.5
Total veg [(b)] (g/day)	-14	-26, -2	0.01	-6	-18, 6	0.3
FV portions[(c)]	-0.3	-0.5 -0.05	0.01	-0.1	-0.3, 0.1	0.4
5-a-day portions[(d)]	-0.2	-0.5, -0.03	0.02	-0.04	-0.3, 0.2	0.7
Total FV[(e)] (g/day)	-24	-43, -6	0.010	-9	-29, 9	0.3
**Nutrients (mg)**						
Vitamin C diet only	-4	-11, 2	0.2	4	-2, 11	0.2
Vitamin C *	-8	-23, 6	0.2	-1	-17, 13	0.8
**Biomarkers**(μmol/l)						
Vitamin C n=717	-4	-7, -0.6	0.020	1	-2, 5	0.3
Total caro[(f)] n= 519	-0.2	-0.4, -0.1	0.003	-0.05	-0.2, 0.1	0.6
α-carotene n= 718	-0.004	-0.01, 0.009	0.5	-0.001	-0.01, 0.01	0.8
β-carotene n= 764	-0.05	-0.1, -0.0006	0.04	0.01	-0.04, 0.07	0.6
Lycopene n= 762	-0.09	-0.1, -0.03	0.003	-0.05	-0.1, 0.01	0.1

For peer review only

Table 1-6 legends

Model 2 adjusted for age, gender, socio-economic status, smoking, ethnicity and food energy. G, gram, CI, Confidence interval, veg, vegetables, mg, milligram, µmol, micromole, l, litre, n, number, FV, fruits and vegetables

a) Total fruit (not including juice) = Fruit(g)+Dried fruit (g)+ Smoothie fruit (g)
b) Total vegetables= Beans (g) + Brassicaceae (g) + Other Vegg + Tomatoes (g) + Tomato Puree (g) +Yellow Red Green (g).
c) FV portions= (Fruit (g) + Driedfruitx3_mean + Tompureex5 mean + beans max mean+ Brassicaceae (g) + Yellow Red Green (g) + Other veg (g) + Tomatoes (g)) / 80 .
d) 5-a-day portions(portions/day)= Fruit veg portions + Fruit juice portions+ Smoothie Fruit portions
e) Total FV (not including juice) = Total fruit +Total vegetables
f) Total carotenoids = Lutein + alpha-cryptoxanthin + beta-cryptoxanthin+ lycopene + alpha-carotene + beta-carotene
*Vitamin C including supplements

For peer review only

STROBE Statement—checklist of items that should be included in reports of observational studies

	Item No	Recommendation	Page
**Title and abstract**	1	(*a*) Indicate the study's design with a commonly used term in the title or the abstract	Pg. 3
		(*b*) Provide in the abstract an informative and balanced summary of what was done and what was found	Pg. 3
**Introduction**			
Background/rationale	2	Explain the scientific background and rationale for the investigation being reported	Pg. 4
Objectives	3	State specific objectives, including any prespecified hypotheses	Pg. 4
**Methods**			
Study design	4	Present key elements of study design early in the paper	Pg. 3
Setting	5	Describe the setting, locations, and relevant dates, including periods of recruitment, exposure, follow-up, and data collection	Pg. 4-5
Participants	6	(*a*) *Cohort study*—Give the eligibility criteria, and the sources and methods of selection of participants. Describe methods of follow-up<br>*Case-control study*—Give the eligibility criteria, and the sources and methods of case ascertainment and control selection. Give the rationale for the choice of cases and controls<br>*Cross-sectional study*—Give the eligibility criteria, and the sources and methods of selection of participants	Fig. 1
		(*b*) *Cohort study*—For matched studies, give matching criteria and number of exposed and unexposed<br>*Case-control study*—For matched studies, give matching criteria and the number of controls per case	NA
Variables	7	Clearly define all outcomes, exposures, predictors, potential confounders, and effect modifiers. Give diagnostic criteria, if applicable	Pg. 4-6
Data sources/ measurement	8*	For each variable of interest, give sources of data and details of methods of assessment (measurement). Describe comparability of assessment methods if there is more than one group	Pg. 4-6
Bias	9	Describe any efforts to address potential sources of bias	NA
Study size	10	Explain how the study size was arrived at	Fig. 1
Quantitative variables	11	Explain how quantitative variables were handled in the analyses. If applicable, describe which groupings were chosen and why	Pg. 5-6
Statistical methods	12	(*a*) Describe all statistical methods, including those used to control for confounding	Pg. 6
		(*b*) Describe any methods used to examine subgroups and interactions	NA
		(*c*) Explain how missing data were addressed	Pg. 7
		(*d*) *Cohort study*—If applicable, explain how loss to follow-up was addressed<br>*Case-control study*—If applicable, explain how matching of cases and controls was addressed<br>*Cross-sectional study*—If applicable, describe analytical methods taking account of sampling strategy	Pg. 6-7
		(*e*) Describe any sensitivity analyses	Pg. 6

Continued on next page

For peer review only - http://bmjopen.bmj.com/site/about/guidelines.xhtml

Results			Page
Participants	13*	(a) Report numbers of individuals at each stage of study—eg numbers potentially eligible, examined for eligibility, confirmed eligible, included in the study, completing follow-up, and analysed	Fig. 1
		(b) Give reasons for non-participation at each stage	NA
		© Consider use of a flow diagram	Fig. 1
Descriptive data	14*	(a) Give characteristics of study participants (eg demographic, clinical, social) and information on exposures and potential confounders	Pg. 7-8
		(b) Indicate number of participants with missing data for each variable of interest	NA
		(c) *Cohort study*—Summarise follow-up time (eg, average and total amount)	NA
Outcome data	15*	*Cohort study*—Report numbers of outcome events or summary measures over time	NA
		Case-control study—Report numbers in each exposure category, or summary measures of exposure	NA
		Cross-sectional study—Report numbers of outcome events or summary measures	Pg. 8
Main results	16	(*a*) Give unadjusted estimates and, if applicable, confounder-adjusted estimates and their precision (eg, 95% confidence interval). Make clear which confounders were adjusted for and why they were included	Pg. 9-10
		(*b*) Report category boundaries when continuous variables were categorized	Pg. 5-6
		(*c*) If relevant, consider translating estimates of relative risk into absolute risk for a meaningful time period	NA
Other analyses	17	Report other analyses done—eg analyses of subgroups and interactions, and sensitivity analyses	Pg. 11
**Discussion**			
Key results	18	Summarise key results with reference to study objectives	Pg. 12
Limitations	19	Discuss limitations of the study, taking into account sources of potential bias or imprecision. Discuss both direction and magnitude of any potential bias	Pg.12-14
Interpretation	20	Give a cautious overall interpretation of results considering objectives, limitations, multiplicity of analyses, results from similar studies, and other relevant evidence	Pg.12-14
Generalisability	21	Discuss the generalisability (external validity) of the study results	Pg. 12-14
**Other information**			
Funding	22	Give the source of funding and the role of the funders for the present study and, if applicable, for the original study on which the present article is based	Pg.15

*Give information separately for cases and controls in case-control studies and, if applicable, for exposed and unexposed groups in cohort and cross-sectional studies.

Note: An Explanation and Elaboration article discusses each checklist item and gives methodological background and published examples of transparent reporting. The STROBE checklist is best used in conjunction with this article (freely available on the Web sites of PLoS Medicine at http://www.plosmedicine.org/, Annals of Internal Medicine at http://www.annals.org/, and Epidemiology at http://www.epidem.com/). Information on the STROBE Initiative is available at www.strobe-statement.org.

For peer review only

BMJ Open

The relationship between sleep duration and fruit/vegetable intake in UK adults: a cross-sectional study from The National Diet and Nutrition Survey.

Journal:	*BMJ Open*
Manuscript ID	bmjopen-2017-020810.R1
Article Type:	Research
Date Submitted by the Author:	23-Jan-2018
Complete List of Authors:	Noorwali, Essra; University of Leeds Faculty of Biological Sciences,<br>Cade, Janet; University of Leeds, School of Food Science<br>Burley, Victoria; University of Leeds, School of Food Science and Nutrition<br>Hardie, Laura; University of Leeds, Leeds Institute of Cardiovascular and Metabolic Medicine, School of Medicine
**Primary Subject Heading**:	Epidemiology
Secondary Subject Heading:	Nutrition and metabolism, Public health
Keywords:	EPIDEMIOLOGY, PUBLIC HEALTH, NUTRITION & DIETETICS, SLEEP MEDICINE

SCHOLARONE™
Manuscripts

For peer review only

The relationship between sleep duration and fruit/vegetable intake in UK adults: a cross-sectional study from The National Diet and Nutrition Survey.

Essra A. Noorwali [(1)] [(2)], Janet E. Cade [(1)], Victoria J. Burley [(1)], Laura J. Hardie [(3)]

[(1)] Nutritional Epidemiology Group, School of Food Science and Nutrition, University of Leeds, Leeds LS2 9JT, UK, [(2)] Department of Clinical Nutrition, Faculty of Applied Medical Sciences, Umm Al-Qura University, P.O. Box 715, Makkah 21955, Saudi Arabia, [(3)] Division of Epidemiology and Biostatistics, Leeds Institute of Cardiovascular and Metabolic Medicine, School of Medicine, University of Leeds, Leeds, LS2 9JT, UK

Correspondence author: Essra A. Noorwali, fsean@leeds.ac.uk or eanoorwali@uqu.edu.sa

For peer review only - http://bmjopen.bmj.com/site/about/guidelines.xhtml

Abstract

Objectives: There is increasing evidence to suggest an association between sleep and diet. The aim of the present study was to examine the association between sleep duration and fruit/vegetable (FV) intakes and their associated biomarkers in UK adults.

Design: cross-sectional.

Setting: data from The National Diet and Nutrition Survey.

Participants: 1612 adults aged 19-65 years were included, pregnant/breastfeeding women were excluded from the analyses.

Outcome measures: Sleep duration was assessed by self-report and diet was assessed by 4-day food diaries, disaggregation of foods containing FV into their components was conducted to determine total FV intakes. Sleep duration was divided to: short (<7 h/d), reference (7-8 h/d) and long (>8 h/d) sleep periods. Multiple regression adjusting for confounders was used for analyses where sleep duration was the exposure and FV intakes and their associated biomarkers were the outcomes. Restricted cubic spline models were developed to explore potential non-linear associations.

Results: In adjusted models , Long Sleepers (LS) consumed on average 28 (95%CI -50,-6, p=0.01) g/d less of total FV compared to Reference Sleepers (RS), whereas Short Sleepers (SS) consumed 24 g/d less (95%CI -42,-6, p=0.006) and had lower levels of FV biomarkers (total carotenoids, β-carotene and lycopene) compared to RS. Restricted cubic spline models showed that the association between sleep duration and FV intake was non-linear (p<0.001) with RS having the highest intakes compared to SS and LS. The associations between sleep duration and plasma total carotenoids (p=0.0035), plasma vitamin C (p=0.009) and lycopene (p<0.001) were non-linear with RS having the highest levels.

Conclusions: These findings show a link between sleep duration and FV consumption. This may have important implications for lifestyle and behaviour change policy.

Strengths and limitations of this study

- The four-day estimated diary has been validated against several biomarkers and demonstrated better estimates of average intakes compared to other dietary assessment methods.
- The disaggregation of foods containing FV into their components helped in assessing total FV intake.
- The self-report of sleep duration was based on memory and could cause over-reporting.
- Cross-sectional studies do not detect causal relationships but only represent associations.

For peer review only - http://bmjopen.bmj.com/site/about/guidelines.xhtml

INTRODUCTION

The consumption of fruits and vegetables has shown to improve the overall health [1] and reduce the risk of chronic diseases[2-4] when 400 grams or more are consumed as recommended by The World Health Organization[5].Hence, identifying lifestyle factors associated with higher intakes of FV is a public health priority.

The relationship between sleep duration and risk of obesity was reported in a recent meta-analysis with short sleep duration associated with a 45% increased risk of obesity due to several behavioural mechanisms including the reduced intake of FV[6]. Thus, it is essential to study FV consumption in relation to sleep duration.

There are limited studies assessing the association between sleep duration and FV consumption in UK adults using validated and detailed dietary data [7]. To our knowledge, this study is the first to use data that disaggregated foods containing FV into their components which helped in assessing total FV intake. Therefore, this study aims to assess the relationship between sleep duration and daily FV consumption and their associated biomarkers in adults aged 19-65 years using data of the National Diet and Nutrition Survey (years 1-4) that represents the UK population.

METHODS

Study population

The National Diet and Nutrition Survey (NDNS) is a government-commissioned rolling programme that started in 1992 to assess the diet, nutrient intake and nutritional status of the UK population[8]. This study used combined data from years 1, 2, 3 and 4 of the rolling programme (2008/09 – 2011/12) for adults aged 19-65 years old [9]. Between April 2008 and March 2011, random samples of 21,573 addresses from 799 postcode sectors were drawn from the UK Postcode Address File. Households were selected randomly and within the household either one adult (aged 19 years and over) and one child (aged 1.5 to 18 years), or one child were randomly selected to participate[8].

Dietary records

The NDNS survey assessed dietary intake using a four-day estimated diary that included instructions on how to complete the diary, as described in detail elsewhere[8]. Participants were asked to record food and drink consumed both at home and away from home for four consecutive days. Participants were asked to record portion sizes as instructed or in

For peer review only - http://bmjopen.bmj.com/site/about/guidelines.xhtml

household measures. They were asked to record brand names, ingredients and quantities, cooking methods, leftovers and dietary supplements**.** Dietary intake was calculated by trained coders and editors in the Diet In Nutrients Out dietary assessment system which calculates food and beverage nutrient intake based on data for >6,000 foods. Detailed information on data coding is provided elsewhere[8].

Fruit and vegetable intake

To determine the total intakes of FV, disaggregation of foods containing FV into their components was conducted by NDNS. FV content of soft drinks, confectionery, cakes (including fruit cake) and biscuits, sugar preserves (including jam) and sweet spreads, savoury snacks and ice cream were excluded from the estimates because they fell into the high fat/high sugars segment of the eat well plate [10]. The disaggregation process and the calculation of 5-a-day portions using disaggregated data is described elsewhere [8 ,11].

Blood Sampling (Fruit and vegetable biomarkers)

Samples were collected between February 2008 and July 2012; Years 1 to 4 of the NDNS Rolling Programme. In Year 1 there was a two week time lag between the start of the interviewer and nurse stages. From Year 2 onwards, the gap was extended, to an average of eight weeks, with the aim of increasing nurse stage response rates. Participants were asked a series of screening questions prior to venepuncture to assess their eligibility for giving a blood sample. Participants with a bleeding or clotting disorder or those taking anti-coagulant medications were excluded from providing a blood sample. The blood taking procedures including collection, processing, analysing and quality control of the blood samples is explained in further detail elsewhere[8]. This study considered available biomarker measurements related to FV consumption namely plasma vitamin C, total carotenoids, α-carotene, β-carotene and lycopene. The detailed procedure for vitamin C and carotenoid analysis is described elsewhere[8].

Sleep Duration

Participants were asked about sleep duration in the following form for week nights and weekends by using a computer assisted personal interview program:

"Over the last seven days, that is since (date) how long did you usually sleep for on weeknights, that is, Sunday to Thursday nights?"

"And over the last seven days, how long did you usually sleep for on a weekend that is Friday and Saturday nights?"

For peer review only - http://bmjopen.bmj.com/site/about/guidelines.xhtml

An average time per night was sought and if respondents worked on night shifts during the last two weeks/weekends, the average time slept during the day should be entered. For this study, two separate variables were generated for sleep duration based on weekdays and weekends for all adults aged 19-65 from year 1-4 in NDNS. Average sleep duration for weekdays and weekends was calculated using the following equation ((minutes slept during the week* 5) + (minutes slept during weekends *2))/7. Sleep duration was categorised based on the literature [12-14] to SS (<7 hours (420 minutes)), RS (7-8 hours (≥ 420 minutes and ≤ 480 minutes)) and LS (> 8 hours (>480 minutes)).

Statistical analysis

Descriptive statistics such as means and proportions were conducted to describe adults from the NDNS according to sleep duration categories. P values of < 0.05 represent statistical significance. Multiple regression analysis was used to assess the relationship between sleep duration, FV intake and biomarkers. Model 1 included adjustment for age and gender only whereas model 2 was adjusted for potential confounders that were identified after the development of a directed acyclic graph these were age, gender, socio-economic status (SES) assessed by National Statistics Socio-economic Classification including 8 categories[15], smoking status [16-19] (current, ex-smoker and never), ethnicity (white, non-white) and energy intake from food. In all analyses, sleep duration was used as the exposure and FV intakes and biomarkers were the outcomes.

We used restricted cubic splines to model non-linear relationships between sleep duration as a continuous exposure (h/day) and total FV intakes as the outcomes (g/d). The splines comprised 4 polynomial segments separated by 5 knots (at the following percentiles of sleep duration 5, 27.5, 50, 72.5 and 95 as recommended by Harrell[20]) with linear regions before the first knot and after the last. The splines for biomarkers comprised 2 polynomial segments separated by 3 knots due to the small number of samples (at the following percentiles of sleep duration 10, 50 and 90 as recommended by Harrell [20]).

Sensitivity analyses were conducted including 1)considering weekdays and weekends separately; and separate analyses were conducted after 2)excluding participants who consumed vitamins, minerals or/and supplements in the previous year (526 participants); 3)excluding those who self-reported currently having a longstanding illness (see supplementary material for included illnesses) (547 participants); 4) excluding those taking prescribed medicines (566 participants) 5)excluding those who reported being vegetarian (39

For peer review only - http://bmjopen.bmj.com/site/about/guidelines.xhtml

participants) 6)including body mass index (BMI) and physical activity as an additional adjustment to the potential confounders in model 2, 6)stratifying the analyses between sleep duration and FV intake by BMI. Statistical analyses were conducted using IC Stata 12 / 13 statistical software[21], missing data were automatically dropped.

RESULTS

General characteristics of NDNS adult participants aged 19-65 years according to sleep duration category are shown in Table 1. Eighty participants were excluded from the analyses due to lack of sleep data or pregnancy/breastfeeding (Fig 1). The 1612 adults included in the study had a mean age of 43 years (95%CI 43, 44) and a mean BMI of 25 (95%CI 25, 26). 33% (n=539) of the participants were SS, 49% of the participants (n= 788) were RS and 18% (n=285) of the participants were LS. In total, 57% (95%CI 55, 60) of the participants were female, 90% (95%CI 89, 92) were white, 46% (95%CI 43, 49) reported taking prescribed medicines and 54% (95%CI 52, 57) never smoked.

Concerning FV consumption, 35% (95%CI 31, 38) of RS consumed 5 or more portions/day of FV whereas 25% (95%CI 21, 31) of LS and 28% (95%CI 24, 32) of SS consumed 5 or more portions of FV/day. LS consumed a mean of 250 (95%CI 233,267) g/d of total FV, RS consumed a mean of 309 (95%CI 297,322) g/d of total FV whereas SS had a mean intake of 276 (95%CI 261, 291) g/d of total FV (Table 1).

For peer review only

Table 1. General characteristics of adults from the NDNS years 1-4 according to sleep duration category.

Characteristics	Sleep Categories <7 hours/day (SS)	7-8 hours/day (RS)	> 8 hours/day (LS)	Total
**Observations (n)**	539	788	285	1612
	**Mean (95%CI)**	**Mean (95%CI)**	**Mean (95%CI)**	**Mean (95%CI)**
**Age (Years)**	45 (44, 46)	44 (43, 45)	39 (38 ,40)	43 (43,44)
**BMI**	26 (25,27)	25 (25,26)	24 (23, 25)	25 (25,26)
**Food energy**	1712 (1665, 1758)	1769 (1731,1807)	1645 (1587,1703)	1727 (1701,1752)
**Equivalised household income**	33K (31K, 35K)	34K (32K, 36K)	29K (26K,32K)	33K (32K, 34K)
**Fruit (g/d)**	98 (89, 106)	115 (107, 124)	82 (73, 92)	103 (98, 108)
**Vegetables (g/d)**	178 (168, 188)	194 (187, 201)	168 (157, 180)	185 (180, 190)
**Total FV (g/d)**	276 (261, 291)	309 (297, 322)	250 (233, 267)	287 (279, 296)
**Plasma vitamin C (µmol/l)**	48 (45, 51)	53 (51, 55)	56 (53, 59)	51 (50, 53)
**Plasma total carotenoids (µmol/l)**	2.2 (2.1, 2.4)	2.5 (2.4, 2.7)	2.4 (2.2 ,2.7)	2.4 (2.3, 2.5)
**Plasma Lycopene (µmol/l)**	0.62 (0.57, 0.67)	0.73 (0.69 , 0.77)	0.69 (0.61,0.76)	0.69 (0.66 , 0.72)
	**% (95%CI)**	**% (95%CI)**	**% (95%CI)**	**% (95%CI)**
**Sex (Female)**	55 (51, 59)	56 (52, 59)	64 (58, 69)	57 (55, 60)
**Ethnicity (White)**	92 (90, 94)	89 (86, 91)	88 (84, 91)	90 (89, 92)
**Has longstanding illness (Yes)**	37 (33, 41)	30 (26, 32)	39 (33, 45)	34 (32, 36)
**Taking prescribed medicine (Yes)**	47 (42, 51)	43 (38, 46)	53 (46, 60)	46 (43,49)
**Employer (Full or part-time employment)**	68 (64, 72)	72 (68, 75)	62 (56, 67)	69 (67, 71)
**SES (Lower managerial and professional)**	28 (24,32)	28 (25,31)	22 (17,27)	27 (24,29)
**Smoking (Never)**	51 (46,55)	57 (53, 60)	54 (48, 60)	54 (52,57)
**Consuming 5 or more portions of FV/day (Yes)**	28 (24, 32)	35 (31, 38)	25 (21, 31)	31 (29, 33)
**Vegetarian (Yes)**	2 (1,3)	3 (2,4)	0.7 (0.1, 2)	2 (1,3)
**Has one child aged between 0-4 years**	15 (12, 18)	13 (10,15)	12 (8,16)	13 (12,15)
**Frequency of drinking alcohol in past 12 months (once or twice a week or month)**	45 (40, 49)	48 (44, 51)	50 (44, 55)	47 (45, 50)

n, number CI, Confidence interval, BMI, Body mass index, SS, short sleepers, RS, reference sleepers, LS, long sleepers, g, gram, d, day, μmol, micromole, l, litre, FV, fruits and vegetables, SES, socio-economic status.

For peer review only - http://bmjopen.bmj.com/site/about/guidelines.xhtml

In adjusted analyses (model 2), SS and LS ate less fruit (g/d), FV portions and total FV (g/d) compared to RS (Table 2). SS ate on average 13 g/d (95%CI -24, -2, p=0.01) less total fruit, 0.2 (95%CI -0.5, -0.06, p=0.01) less portions/d of FV and 24 g/d (95%CI -42,-6, p=0.006) less total FV. LS consumed on average 16 g/d (95%CI -30, -2, p=0.01) less total fruit, 0.2 (95%CI -0.5, 0.01, p= 0.06) less portions/d of FV and 28 g/d (95%CI -50,-6, p=0.01) less total FV. In model 1 SS had on average 17 g/d (95%CI -29,-5, p=0.004), LS 19 g/d (95%CI -34, -4, p=0.009) less vegetable intake compared to RS but the differences became borderline significant with further adjustment.

In adjusted analyses (model 2), SS had lower levels of plasma FV biomarkers except α-carotene and vitamin C compared to RS. In contrast, LS had higher vitamin C levels compared to RS. LS had 4 μmol/l higher plasma vitamin C (95%CI 0.1,8, p=0.04) compared to RS. SS had 0.2 μmol/l lower plasma total carotenoids (95%CI -0.4, -0.08, p=0.004), 0.05 μmol/l lower plasma β-carotene (95%CI -0.1, -0.009, p=0.01) and 0.08 μmol/l lower plasma lycopene (95%CI -0.1,-0.02, p=0.005) compared to RS. This was confirmed with SS having less intake of tomatoes compared to RS in adjusted models (-5g/d, 95%CI -9, -0.1, p=0.04). SS had a mean intake of 42 g/d (95%CI 38, 46) of tomatoes, RS had 48 g/d (95%CI 45, 51) and LS had 41 g/d (95%CI 36, 46).

For peer review only

For peer review only - http://bmjopen.bmj.com/site/about/guidelines.xhtml

Table 2. The association between sleep duration categories, FV intakes and their biomarkers of adults from the NDNS years 1-4.

Short sleepers (<7h/d) and Long sleepers (>8h/d) compared to the reference group (7-8 h/d)								
**Models**	**Model 1 (n=1612)**				**Model 2 (n= 1610)**			
	**Short sleepers <7 hours/day**		**Long sleepers >8 hours/day**		**Short sleepers <7 hours/day**		**Long sleepers >8 hours/day**	
**FV intake**	**Mean difference(95%CI)**	**P value**	**Mean difference(95%CI)**	**P value**	**Mean difference(95%CI)**	**P value**	**Mean difference(95%CI)**	**P value**
Total fruit[(a)] (g/day)	-19 (-31, -8)	0.001	-24 (-38 ,-10)	0.001	-13 (-24, -2)	0.01	-16 (-30, -2)	0.01
Total veg[(b)] (g/day)	-17 (-29, -5)	0.004	-19 (-34 ,-4)	0.009	-10 (-21, 0.5)	0.06	-11 (-25, 2)	0.09
FV portions[(c)]	-0.4 (-0.6, -0.2)	<0.001	-0.5 (-0.8, -0.1)	0.001	-0.2 (-0.5, -0.05)	0.01	-0.2 (-0.5 ,-0.01)	0.04
5-a-day portions[(d)]	-0.4 (-0.7, -0.2)	<0.001	-0.5 (-0.8, -0.1)	0.002	-0.2 (-0.5, -0.06)	0.01	-0.2 (-0.5, 0.01)	0.06
Total FV[(e)](g/day)	-37 (-56, -18)	<0.001	-44 (-67, -20)	<0.001	-24 (-41, -6)	0.006	-28 (-50, -6)	0.01
**Nutrients (mg/d)**								
Vitamin C diet only	-9 (-16 , -2)	0.01	-10 (-19, -1)	0.02	-5 (-12, 1)	0.1	-4 (-12, 4)	0.3
Vitamin C *	-13 (-27, 0.8)	0.06	-21 (-39, -3)	0.01	-7 (-21, 6)	0.2	-12 (-30,5)	0.1
**Biomarkers (µmol/l)**								
Vitamin C	-4 (-8, -1)	0.006	3 (-1 , 7)	0.1	-2 (-6, 0.3)	0.07	4 (0.1, 8)	0.04
Total carot[(f)]	-0.2 (-0.4, -0.1)	0.002	-0.1 (-0.3, 0.1)	0.3	-0.2 (-0.4, -0.08)	0.004	-0.06 (-0.2 ,0.1)	0.5
α-carotene	-0.01 (-0.02,0.003)	0.1	-0.009 (-0.02 , 0.007)	0.2	-0.006 (-0.01, 0.007)	0.3	-0.004 (-0.02, 0.01)	0.6
β-carotene	-0.07 (-0.1, -.02)	0.003	-0.01 (-0.07 , 0.05)	0.7	-0.05 (-0.1 ,-0.009)	0.01	0.009 (-0.05, 0.07)	0.7
Lycopene	-0.1 (-0.1 ,-0.04)	0.001	-0.06 (-0.1 ,0.01)	0.09	-0.08 (-0.1, -0.02)	0.005	-0.05 (-0.1, 0.02)	0.1

Model 1 adjusted for age and gender
Model 2 adjusted for age, gender, socio-economic status, smoking, ethnicity and food energy
G, gram, CI, Confidence interval, veg, vegetables, mg, milligram, µmol, micromole, l, litre, n, number, FV, fruits and vegetables,*Vitamin C including supplements

a) Total fruit (not including juice) = Fruit(g)+Dried fruit (g)+ Smoothie fruit (g)
b) Total vegetables= Beans (g) + Brassicaceae (g) + Other vegetables (g) + Tomatoes (g) + Tomato Puree (g) +Yellow Red Green (g)
c) FV portions= (Fruit (g) + Driedfruitx3_mean + Tompureex5 mean + beans max mean+ Brassicaceae (g) + Yellow Red Green (g) + Other vegetables (g) + Tomatoes (g))/ 80
d) 5-a-day portions(portions/day)= Fruit/vegetable portions + Fruit juice portions+ Smoothie fruit portions
e) Total FV (not including juice) = Total fruit +Total vegetables
f) Total carotenoids = Lutein + alpha-cryptoxanthin + beta-cryptoxanthin+ lycopene + alpha-carotene + beta-carotene

For peer review only - http://bmjopen.bmj.com/site/about/guidelines.xhtml

Restricted cubic spline modelling (Fig 2) showed that the association between sleep duration and total FV intake (g/d) was non-linear ($p<0.001$) with participants sleeping 7-8h/d having the highest intakes compared to SS and LS. Similarly, the association between sleep duration and plasma vitamin C ($p=0.009$) (Fig 3A), total carotenoids ($p=0.0035$) (Fig 3B) and lycopene ($p<0.001$) (Fig 3C) were non-linear.

Sensitivity analysis

Sensitivity analysis showed broadly similar results (available as supplementary material tables 1-7). Including adjustment for BMI and physical activity in the fully adjusted model did not affect the results. Results of separate analysis excluding participants who consumed minerals, vitamins and/or food supplements, being vegan/vegetarian, having a longstanding illness and consuming prescribed medicines, remained similar with SS consuming less FV in comparison to RS but no difference between LS FV intakes and RS. The association between sleep duration and biomarkers were similar with SS having lower levels compared to RS and LS having higher levels of plasma vitamin C compared to RS. Results dividing the exposure into weekday and weekend sleep duration were similar, SS on average consumed less g/d of FV and had lower levels of biomarkers on weekdays and weekends compared to RS. LS on average consumed less g/d of FV on weekdays compared to RS.

For peer review only - http://bmjopen.bmj.com/site/about/guidelines.xhtml

DISCUSSION

To our knowledge, this is the first nationally representative study to examine the association between sleep duration and FV intakes using disaggregated data among UK adults. The results of this study show SS and LS have lower intakes of FV compared to RS. Results of FV biomarkers show lower levels of all plasma biomarkers except α-carotene and vitamin C in SS compared to RS in contrast to plasma vitamin C levels in LS which were higher than RS. Similar results were noted after further adjustment for BMI and physical activity, excluding participants who had a longstanding illness, consumed prescribed medicines and those who consumed supplements, minerals or/and vitamins in the previous year. The association between sleep duration, FV intake and biomarkers were non-linear with RS having the highest intakes and levels of biomarkers compared to SS and LS as shown in the restricted cubic spline modelling. Thus, these findings suggest that among UK adults RS have the highest intakes of FV compared to SS and LS.

These results are in line with several other cross-sectional studies [12 ,13 ,22-24]. Although the studies differed in sample size, ethnicity, dietary assessment methods and categorisation of sleep duration, the results showed a lower intake of FV in short/long sleepers compared to RS. Women with short and long sleep durations had low intakes of FV in the USA or Puerto Rico[22] which was similar to the results of this study. Additionally, short sleep duration was associated with obesity-related behaviours including low FV consumption in rural communities in Missouri, Tennessee and Arkansas[23]. In a study that examined the association between sleep duration and diet quality among women following childbirth, short sleep duration was not associated with diet quality whereas long sleep duration was associated with lower consumption of total and whole fruit[25]. Katagiri *et al.* (2014) measured the association between sleep quality and diet and noted that poor sleep quality was significantly associated with low intakes of total vegetables, green/yellow vegetables and other vegetables[26]. The study suggested that the relationship of dietary intake with sleep quality is similar to that with sleep duration. Regarding the results of FV biomarkers, our results were supported by two other studies [12 ,27]. Grandner *et al.* (2013) showed a significant lower intakes of lycopene in very short sleepers (<5hours) [27]. Beydoun *et al.* (2014) reported that short sleep duration was associated with lower serum levels of vitamin C, total carotenoids, α-carotene and β-carotene compared to RS[12]. It is unclear why the results of this study observed a higher plasma vitamin C levels in LS however, this may be explained by differences in food variety or misreporting of diet intake[27]. This also could be

For peer review only - http://bmjopen.bmj.com/site/about/guidelines.xhtml

due to biomarkers measuring long term dietary intake while diet intake was assessed by a 4-day food diary.

In contrast, a recent study examined the association between sleep duration and cardiovascular risk behaviours using data from the UK biobank found results which were inconsistent with our own. Long sleep duration was positively associated with vegetable intake[28]. These results which contrast with our findings may be due to the different assessment methods of sleep duration and FV intake. Sleep duration assessment in the UK biobank did not consider weekday and weekend sleep duration separately as conducted in the NDNS study since sleep duration may differ between those days[29]. Furthermore, self-report of sleep duration may differ by question format by reporting short sleep duration when asked a single question [30]. Sleep duration in the UK biobank study was assessed by asking one question in regard to sleep every 24h whereas our study assessed sleep duration by asking two separate questions of sleep based on weeknights and weekends. FV intakes were assessed differently in the UK biobank[28] and the NDNS survey[11]. Patterson *et al.* (2016) assessed FV intake by considering diet intake in the previous year and asking how many pieces of fresh fruit would participants eat per day and how many heaped table-spoons of vegetables participants would eat on average per day. This method was based on the UK guidelines that a portion of vegetables is three heaped tablespoons whereas the NDNS survey assessed dietary intake using a four-day estimated diary and disaggregated foods containing FV which is considered a better estimate of average intakes compared to other dietary assessment methods[31]. Additionally, this study conducted supportive biomarker analyses. In a home-based intervention study that assessed the effects of extended bedtimes on sleep duration and food desire, desire for FV was not affected by added sleep[32]. However, the study had several limitations including a small sample size which may limit the generalizability to more diverse populations. One of the major limits of the intervention study [32] is the short duration of intervention (2 weeks) which does not measure the potential effects over a longer period. Experimental studies differ from free-living individuals therefore; it is required to consider the potential for non-representative samples taking part in experimental studies. Furthermore, the association between FV intake and sleep duration was assessed among American pregnant women. Total daily FV consumption was not associated with sleep duration[33]. This could be due to the different sample and dietary assessment methods. FV was assessed by asking women how many times per day, week or month they consumed FV.

For peer review only - http://bmjopen.bmj.com/site/about/guidelines.xhtml

Several potential mechanisms may underlie the association between sleep duration and diet intake [7 ,34 ,35]. Short sleep duration or disrupted sleep may lead to emotional stress, impaired decision making, and increased reward sensitivity to calorie-dense foods and lower FV intake. Changes in appetite hormones, ghrelin and leptin, due to lack/disrupted sleep may increase the preference for energy-dense foods leading to lower intakes of FV. Although potential mechanisms were not measured in this study, they may be the underlying reasons for decreased intake of FV in SS and LS. On the other hand, sleep may be promoted by foods such as kiwifruits, tart cherries, milk and chamomile tea for their impact on tryptophan availability and the synthesis of serotonin and melatonin[36]. This provides insight to the relationship between sleep and diet being potentially bi-directional. Future interventional trials are required to incorporate objective measures of sleep to clarify the relationships between sleep and FV intakes. Sleep extension intervention has been reported to reduce the intakes of free sugars in a 4-week randomised controlled pilot trial[37]. Longer term, fully powered sleep extension studies on FV intake and their associated biomarkers are needed to confirm these results.

Strengths and limitations of the study

The main strength of this study was the disaggregation of foods containing FV into their components which helps in assessing total FV intake[11]. Furthermore, the four-day estimated diary has been validated against several biomarkers and demonstrated better estimates of average intakes compared to other dietary assessment methods.

This study has several limitations including the self-report of sleep duration which was based on memory and could cause over-reporting[38]. Further limitations include lack of consideration of other sleep factors such as sleep quality[26], sleep timing [39], sleep problems, typical week information , shift-work and chronotype[28]. In Year 1, weekend days were oversampled and in year 2 they were under-sampled to redress that however, in the years 1 to 4 combined data there still remains a slightly higher proportion of weekend days. Eating habits vary between weekdays and weekends [40] which could lead to a bias in the reporting of FV intake. The small number of participants in the obtained biomarkers was a further limitation. The association between sleep duration and FV intake is a bi-directional relationship and the causal pathways underlying the relationship cannot be detected in cross-sectional studies[26].

PUBLIC HEALTH IMPLICATIONS

For peer review only - http://bmjopen.bmj.com/site/about/guidelines.xhtml

Sleep duration among UK adults has been declining recently with 70% of UK adults sleeping less than 7h/night according to the Sleep Council [41]. Additionally, the intake of FV is decreasing among UK adults with only 30% of them meeting the 5-a-day recommendation according to the NDNS results provided by Public Health England[8]. If the results of this study were confirmed in prospective and interventional studies this would highlight the importance of translating the scientific evidence focusing on the relationship between sleep and diet into practical messages that can help the public to prevent chronic diseases. This would include making different populations aware of the relationship between sleep and diet by providing more information on sleep in national dietary guidelines to enhance healthy lifestyle recommendations. In addition, this information can be incorporated in hospitals to educate healthcare professionals, weight-loss programs and other programs targeting improvement in overall health. This information is also essential for those caring for at risk groups such as the elderly and those with chronic diseases[42].

CONCLUSIONS

The results of this study suggest a link between sleep duration and FV intake. Sleep duration was non-linearly related to self-reported FV intakes and their associated biomarkers with RS having the highest intakes of FV and levels of associated biomarkers compared to SS and LS. These results may have important implications for lifestyle and behaviour change policy.

For peer review only

For peer review only - http://bmjopen.bmj.com/site/about/guidelines.xhtml

ACKNOWLEDGMENTS

We thank the study participants and the UK Data Service.

DATA AVAILABILITY

All National Diet and Nutrition Survey Data are available online at the UK Data Service website (see https://www.gov.uk/government/collections/national-diet-and-nutrition-survey).

Ethical approval was not required for this study.

FINANCIAL SUPPORT

Essra Noorwali is in receipt of a scholarship from Umm Al-Qura University, Makkah, Saudi Arabia.

CONFLICT OF INTEREST

None.

AUTHORSHIP

The authors' contributions are as follows: Essra Noorwali was the principal investigator and contributed to the study design, data analyses, interpretation of the findings and wrote the manuscript; Victoria Burley contributed to the study design, data analyses, interpretation of findings; Laura Hardie; contributed to the study design, data analyses interpretation of findings and article revision; Janet Cade contributed to interpretation of findings and article revision. All authors read and approved the final version of the manuscript.

For peer review only

REFERENCES

1. Wang X, Ouyang, Y., Liu, J., et al. Fruit and vegetable consumption and mortality from all causes, cardiovascular disease, and cancer: systematic review and dose-response meta-analysis of prospective cohort studies. Bmj 2014;**349**:g4490.

2. Ishihara J UM. Relationship Between Vegetables and Fruits (Antioxidant Vitamins, Minerals, and Fiber) Intake and Risk of Cardiovascular Disease. Reference Module in Biomedical Sciences 2017.

3. Liu S, Manson, J.E., Lee, I.M., et al. Fruit and vegetable intake and risk of cardiovascular disease: the Women's Health Study. The American journal of clinical nutrition 2000;**72**(4):922-28.

4. Aune D, Giovannucci E., Boffetta P., et al. Fruit and vegetable intake and the risk of cardiovascular disease, total cancer and all-cause mortality–a systematic review and dose-response meta-analysis of prospective studies. Int J Epidemiol 2016.

5. WHO Study Group on Diet Nutrition and Prevention of Noncommunicable Diseases. report of a WHO Study Group. Technical report series. Geneva, 1990:203p.

6. Wu Y, Zhai L, Zhang D. Sleep duration and obesity among adults: a meta-analysis of prospective studies. Sleep Med 2014;**15**(12):1456-62.

7. Dashti HS, Scheer FA, Jacques PF, et al. Short sleep duration and dietary intake: epidemiologic evidence, mechanisms, and health implications. Adv Nutr 2015;**6**(6):648-59.

8. Bates B, Lennox A, Prentice A, et al. National Diet and Nutrition Survey Results from Years 1, 2, 3 and 4 (combined) of the Rolling Programme (2008/2009-2011/2012) 2014:Availablefrom:https://www.gov.uk/government/uploads/system/uploads/attachment_data/file/594360/NDNS_Y1_to_4_UK_report_executive_summary_revised.pdf

9. NatCen Social Research, MRC Elsie Widdowson Laboratory, University College London (2017). Medical School. National Diet and Nutrition Survey Years 1-6, 2008/09-2013/14. [data collection]. 8th Edition.UK Data Service, [Accessed 24 April 2017]. Available from: http://doi.org/10.5255/UKDA-SN-6533-7.

10. Eat well plate. 2013. http://www.nhs.uk/Livewell/Goodfood/Pages/eatwell-plate.aspx.[Accessed September 2016]

11. Fitt E, Mak TN, Stephen AM, et al. Disaggregating composite food codes in the UK National Diet and Nutrition Survey food composition databank. Eur J Clin Nutr 2010;**64 Suppl 3**:S32-6.

12. Beydoun MA, Gamaldo AA, Canas JA, et al. Serum nutritional biomarkers and their associations with sleep among US adults in recent national surveys. PLoS One 2014;**9**(8):e103490.

13. Haghighatdoost F, Karimi G, Esmaillzadeh A, et al. Sleep deprivation is associated with lower diet quality indices and higher rate of general and central obesity among young female students in Iran. Nutrition 2012;**28**(11-12):1146-50.

14. Cassidy S, Chau JY, Catt M, et al. Cross-sectional study of diet, physical activity, television viewing and sleep duration in 233,110 adults from the UK Biobank; the behavioural phenotype of cardiovascular disease and type 2 diabetes. BMJ Open 2016;**6**(3):e010038.

For peer review only - http://bmjopen.bmj.com/site/about/guidelines.xhtml

15. The National Statistics Socio-economic classification (NS-SEC rebased on SOC2010). http://webarchive.nationalarchives.gov.uk/20160105160709/http://www.ons.gov.uk/ons/guide-method/classifications/current-standard-classifications/soc2010/soc2010-volume-3-ns-sec--rebased-on-soc2010--user-manual/index.html.[Accessed September 2016].

16. Zhang L SJ, Caffo B, Punjabi NM. Cigarette smoking and nocturnal sleep architecture. Am J Epidemiol 2006;**15**(164(6)):529-37.

17. Jaehne A UT, Feige B, et al. How smoking affects sleep: a polysomnographical analysis. Sleep medicine 2012;**31**(13(10)):1286-92.

18. Lohse Tina SR, Matthias Bopp, et al. Heavy Smoking Is More Strongly Associated with General Unhealthy Lifestyle than Obesity and Underweight. PloS one 2016;**11**(2):e0148563.

19. Palaniappan U SL, O'Loughlin J, Gray-Donald K. . Fruit and vegetable consumption is lower and saturated fat intake is higher among Canadians reporting smoking. J Nutr 2001;**131**(7):1952-8.

20. Harrell F. *Regression Modeling Strategies : With Applications to Linear Models, Logistic Regression, and Survival Analysis* 1st ed. New York Springer, 2001.

21. StataCorp. 2013. Stata Statistical Software: Release 13. College Station, TX: StataCorp LP.

22. Kim S, DeRoo LA, Sandler DP. Eating patterns and nutritional characteristics associated with sleep duration. Public Health Nutr 2011;**14**(5):889-95.

23. Stamatakis KA, Brownson RC. Sleep duration and obesity-related risk factors in the rural Midwest. Prev Med 2008;**46**(5):439-44.

24. Mossavar-Rahmani Y, Jung M, Patel SR, et al. Eating behavior by sleep duration in the Hispanic Community Health Study/Study of Latinos. Appetite 2015;**95**:275-84.

25. Xiao RS, Moore Simas TA, Pagoto SL, et al. Sleep Duration and Diet Quality Among Women Within 5 Years of Childbirth in the United States: A Cross-Sectional Study. Matern Child Health J 2016.

26. Katagiri R, Asakura K, Kobayashi S, et al. Low intake of vegetables, high intake of confectionary, and unhealthy eating habits are associated with poor sleep quality among middle-aged female Japanese workers. J Occup Health 2014;**56**(5):359-68.

27. Grandner MA, Jackson N, Gerstner JR, et al. Dietary nutrients associated with short and long sleep duration. Data from a nationally representative sample. Appetite 2013;**64**:71-80.

28. Patterson F, Malone SK, Lozano A, et al. Smoking, Screen-Based Sedentary Behavior, and Diet Associated with Habitual Sleep Duration and Chronotype: Data from the UK Biobank. Ann Behav Med 2016; 50(5) :715-726.

29. Paine SJ, Gander PH. Differences in circadian phase and weekday/weekend sleep patterns in a sample of middle-aged morning types and evening types. Chronobiol Int 2016;**33**(8):1009-17.

30. Lauderdale DS. Survey Questions About Sleep Duration: Does Asking Separately About Weekdays and Weekends Matter? Behav Sleep Med 2014;**12**((2)):158-68.

For peer review only - http://bmjopen.bmj.com/site/about/guidelines.xhtml

31. Day NE, McKeown N, Wong MY, et al. Epidemiological assessment of diet: a comparison of a 7-day diary with a food frequency questionnaire using urinary markers of nitrogen, potassium and sodium. Int J Epidemiol 2001;**30**(2):309-17.

32. Tasali E, Chapotot F, Wroblewski K, et al. The effects of extended bedtimes on sleep duration and food desire in overweight young adults: a home-based intervention. Appetite 2014;**80**:220-4.

33. Duke CH, Williamson JA, Snook KR, et al. Association Between Fruit and Vegetable Consumption and Sleep Quantity in Pregnant Women. Maternal child health journal 2017; 21(5) : 966-973.

34. Lundahl A, Nelson TD. Sleep and food intake: A multisystem review of mechanisms in children and adults. J Health Psychol 2015;**20**(6):794-805.

35. Chaput JP. Sleep patterns, diet quality and energy balance. Physiol Behav 2014;**134**:86-91.

36. Peuhkuri K SN, Korpela R Diet promotes sleep duration and quality. Nutr Res 2012; **32(5)**:309-19.

37. Al Khatib H, Hall WL,Creedon A,Ooi E,Masri T, Harding SV, McGowan L, Darzi J, Pot GK.Sleep extension is a feasible lifestyle intervention in free-living adults who are habitually short sleepers: a potential strategy for decreasing intake of free sugars? A randomized controlled pilot study. The American Journal of Clinical Nutrition 2018: nqx030.

38. Lauderdale DS, Knutson KL, Yan LL, et al. Self-reported and measured sleep duration: how similar are they? Epidemiology 2008;**19**(6):838-45.

39. Golley RK, Maher CA, Matricciani L, et al. Sleep duration or bedtime? Exploring the association between sleep timing behaviour, diet and BMI in children and adolescents. Int J Obes (Lond) 2013;**37**(4):546-51.

40. Decastro JM. Social Facilitation of the Spontaneous Meal Size of Humans Occurs on Both Weekdays and Weekends. Physiol Behav 1991;**49**(6):1289-91.

41. The Sleep Council.The Great British bedtime report. http://www.sleepcouncil.org.uk/wp-content/uploads/2013/02/The-Great-British-Bedtime-Report.pdf (2013) accessed 26/05/2017.

42. Frank S,Gonzalex, K, Lee-Ang L, et al.Diet and Sleep Physiology: Public Health and Clinical Implications. Frontiers in Neurology 2017;**8**(393).

For peer review only

For peer review only - http://bmjopen.bmj.com/site/about/guidelines.xhtml

Figure 1. Participants' flow chart

Figure 2. The association between sleep duration and FV intake from the restricted cubic spline modelling. Black lines plot the predicted FV intakes with 95% confidence interval (grey shaded area) for typical participants (females, white, never smokers, lower managerial and professional occupation, using mean age (43.1) and mean food energy (1727.05)).

Figure 3. The association between sleep duration and FV biomarkers from the restricted cubic spline modelling. Black lines plot the predicted FV biomarkers values **A)** vitamin C **B)** Total carotenoids **C)** lycopene with 95% confidence interval (grey shaded area) for typical participants (females, white, never smokers, lower managerial and professional occupation, using mean age (43.1) and mean food energy (1727.05)).

For peer review only

For peer review only - http://bmjopen.bmj.com/site/about/guidelines.xhtml

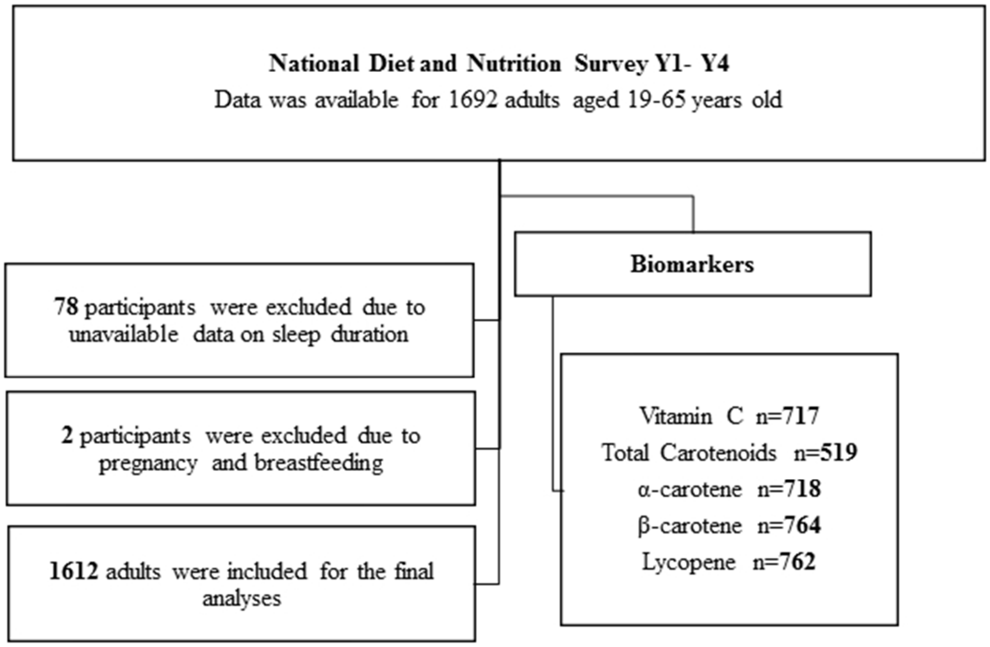

Participants' flow chart

53x38mm (300 x 300 DPI)

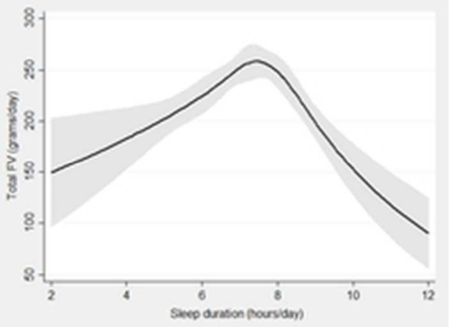

The association between sleep duration and FV intake from the restricted cubic spline modelling. Black lines plot the predicted FV intakes with 95% confidence interval (grey shaded area) for typical participants (females, white, never smokers, lower managerial and professional occupation, using mean age (43.1) and mean food energy (1727.05)).

18x13mm (300 x 300 DPI)

For peer review only

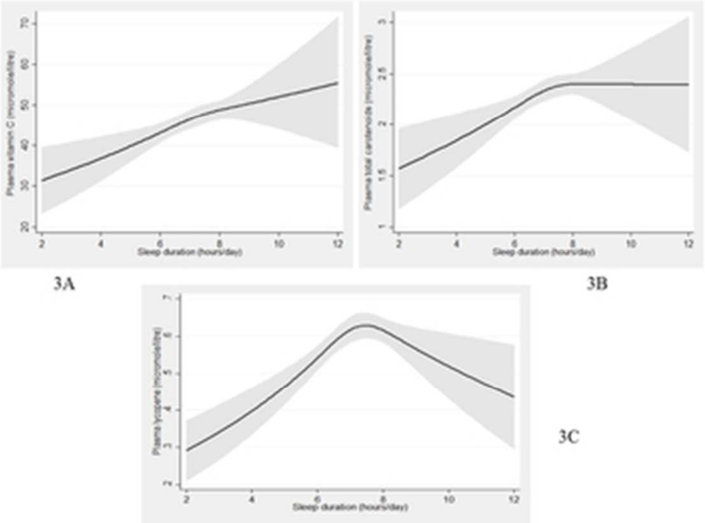

The association between sleep duration and FV biomarkers from the restricted cubic spline modelling. Black lines plot the predicted FV biomarkers values A) vitamin C B) Total carotenoids C) lycopene with 95% confidence interval (grey shaded area) for typical participants (females, white, never smokers, lower managerial and professional occupation, using mean age (43.1) and mean food energy (1727.05)).

30x22mm (300 x 300 DPI)

For peer review only - http://bmjopen.bmj.com/site/about/guidelines.xhtml

Online supplementary material

Table 1. The association between sleep duration categories and FV intakes and associated biomarkers for adults from the NDNS years 1-4 after excluding participants who consume prescribed medicines.

	Sleep categories compared to the reference group (7-8 hours/day)			
**Model 2 (n=1044)**	**Short sleepers <7 hours/day**		**Long sleepers >8 hours/day**	
**FV intake**	**Mean difference (95%CI)**	**P value**	**Mean difference (95%CI)**	**P value**
Total fruit(a) (g/day)	-13 (-27, -0.4)	0.04	-17 (-34, -0.1)	0.04
Total veg(b) (g/day)	-10 (-24, 4)	0.1	-5 (-24, 2)	0.5
FV portions(c)	-0.2 (-0.5, -.005)	0.04	-0.1 (-0.5, 0.1)	0.3
5-a-day portions(d)	-0.2 (-0.5, -0.0003)	0.05	-0.1 (-0.5, 0.1)	0.3
Total FV (e)(g/day)	-24 (-45,-2)	0.03	-23 (-51,4)	0.1
**Nutrients (mg)**				
Vitamin C diet only	-5 (-13, 2)	0.1	-4(-15,5)	0.3
Vitamin C *	-7 (-24, 10)	0.4	-11 (-33, 11)	0.3
**Biomarkers (µmol/l)**				
Vitamin C n= 375	-0.6 (-5, 3)	0.7	5 (-0.09, 11)	0.05
Total caro(f) n= 294	-0.2 (-0.5, -0.02)	0.03	-0.08 (-0.4, 0.2)	0.5
α-carotene n= 378	-0.005 (-0.02,0.01)	0.5	-0.009 (-0.03, 0.01)	0.4
β-carotene n= 402	-0.05 (-0.1, 0.02)	0.1	-0.02(-0.1, 0.07)	0.6
Lycopene n= 403	-0.06 (-0.1, 0.01)	0.1	-0.06 (-0.1, 0.04)	0.2

566 participants reported taking prescribed medicines and were excluded from the analyses.

For peer review only

Table 2. The association between sleep duration categories and FV intakes and associated biomarkers for adults from the NDNS years 1-4 after excluding participants who reported consuming vitamins, minerals and/or supplements in the past year.

		Sleep categories compared to the reference group (7-8hours/day)			
**Model 2 (n=1084)**		**Short sleepers <7 hours/day**		**Long sleepers >8 hours/day**	
**FV intake**		**Mean difference (95%CI)**	**P value**	**Mean difference (95%CI)**	**P value**
Total fruit[(a)] (g/day)		-5 (-17,7)	0.4	-10 (-25, 0.5)	0.2
Total veg[(b)] (g/day)		-11 (-24, 1)	0.09	-7 (-24, 8)	0.3
FV portions[(c)]		-0.1 (-0.4, 0.07)	0.1	-0.1 (-0.4, 0.1)	0.3
5-a-day portions[(d)]		-0.1 (-0.4, 0.09)	0.2	-0.1 (-0.4, 0.2)	0.4
Total FV[(e)](g/day)		-16 (-36, 3)	0.1	-17 (-43,7)	0.1
**Nutrients (mg)**					
Vitamin C diet only		1 (-6, 8)	0.7	2 (-6, 11)	0.5
Vitamin C *		0.2 (-8, 8)	0.9	2 (-7, 13)	0.5
**Biomarkers (µmol/l)**					
Vitamin C	n= 462	-3 (-7, 0.6)	0.09	6 (1, 11)	0.009
Total caro [(f)]	n= 330	-0.2 (-0.4, -0.01)	0.03	0.06 (-0.2 ,0.3)	0.6
α-carotene	n= 461	-0.005 (-0.02,0.009)	0.4	0.0007 (-0.01, 0.02)	0.9
β-carotene	n= 492	-0.05 (-0.1, 0.002)	0.06	0.06 (-0.01,0.1)	0.1
Lycopene	n= 490	-0.08 (-0.1, -0.005)	0.03	-0.04 (-0.1, 0.04)	0.3

526 participants reported taking vitamins, minerals or supplements in the past year and were excluded from the analyses.

For peer review only

Table 3. The association between sleep duration categories and FV intakes and associated biomarkers for adults from the NDNS years 1-4 after excluding those who have a longstanding illness.

		Sleep categories compared to the reference group (7-8hours)			
**Model 2 (n= 1063)**		**Short sleepers <7 hours**		**Long sleepers >8 hours**	
**FV intake**		**Mean difference(95%CI)**	**P value**	**Mean difference (95%CI)**	**P value**
Total fruit[(a)] (g/day)		-15 (-29, -2)	0.02	-9 (-26, 7)	0.2
Total veg[(b)] (g/day)		-14 (-28, -0.04)	0.04	-10 (-28, 8)	0.2
FV portions[(c)]		-0.4 (-0.7, -0.1)	0.004	-0.1 (-0.5, 0.1)	0.2
5-a-day portions[(d)]		-0.4 (-0.7, -0.1)	0.005	-0.1 (-0.5 ,0.1)	0.3
Total FV[(e)](g/day)		-30 (-52, -8)	0.007	-20 (-48, 8)	0.1
**Nutrients (mg)**					
Vitamin C diet only		-5 (-14, 2)	0.1	-1 (-11, 9)	0.8
Vitamin C *		-12 (-30, 5)	0.1	-9 (-32, 14)	0.4
**Biomarkers (μmol/l)**					
Vitamin C	n= 474	-2 (-6, 1)	0.2	4 (-0.4 ,9)	0.07
Total caro [(f)]	n= 369	-0.3 (-0.5, -0.08)	0.007	-0.04 (-0.3,0.2)	0.7
α-carotene	n= 477	-0.006 (-0.02, 0.01)	0.4	0.001 (-0.01, 0.02)	0.9
β-carotene	n= 506	-0.06 (-0.1, -0.003)	0.03	0.01 (-0.06, 0.09)	0.6
Lycopene	n= 505	-0.07 (-0.1, 0.008)	0.08	-0.05 (-0.1, 0.05)	0.3

547 participants reported having a longstanding illness and were excluded from the analyses.

For peer review only

Table 4. The association between sleep duration categories and FV intakes and associated biomarkers for adults from the NDNS years 1-4 after excluding those who reported being vegetarian.

		Sleep categories compared to the reference group (7-8hours/day)			
**Model 2 (n= 1571)**		**Short sleepers <7 hours/day**		**Long sleepers >8 hours/day**	
**FV intake**		**Mean difference(95%CI)**	**P value**	**Mean difference (95%CI)**	**P value**
Total fruit[(a)] (g/day)		-14 (-25,-3)	0.009	-9 (-26,7)	0.2
Total veg[(b)] (g/day)		-11 (-22, 0.1)	0.05	-11 (-25,3)	0.1
FV portions[(c)]		-0.3 (-0.5, -0.07)	0.009	-0.3 (-0.5, -0.01)	0.03
5-a-day portions[(d)]		-0.3 (-0.5,-0.08)	0.008	-0.2 (-0.5, 0.008)	0.05
Total FV [(e)](g/day)		-25 (-43,-8)	0.004	-29 (-51, -7)	0.009
**Nutrients (mg)**					
Vitamin C diet only		-5 (-12, 0.9)	0.09	-4 (-12, 4)	0.3
Vitamin C *		-4 (-18, 8)	0.4	-8 (-25, 8)	0.3
**Biomarkers (µmol/l)**					
Vitamin C	n= 702	-3 (-6, 0.1)	0.06	4 (0.09, 8)	0.04
Total caro [(f)]	n= 506	-0.2 (-0.4, -0.09)	0.003	-0.05 (-0.2, 0.1)	0.6
α-carotene	n= 702	-0.006 (-0.01,0.007)	0.3	-0.002 (-0.01 ,0.01)	0.7
β-carotene	n= 748	-0.05 (-0.1, -0.008)	0.02	0.01 (-0.04, 0.07)	0.6
Lycopene	n= 746	-0.09 (-0.1, -0.03)	0.003	-0.05 (-0.1 0.02)	0.2

39 participants reported being vegetarian and were excluded from this analyses.

For peer review only - http://bmjopen.bmj.com/site/about/guidelines.xhtml
For peer review only

Table 5. The association between sleep duration categories and FV intakes, nutrients and associated biomarkers for adults from the NDNS years 1-4 after further adjusting for BMI and physical activity.

		Sleep categories compared to the reference group (7-8hours/day)			
**Model 2 (n=1171)**		**Short sleepers <7 hours/day**		**Long sleepers >8 hours/day**	
**FV intake**		**Mean difference (95%CI)**	**P value**	**Mean difference (95%CI)**	**P value**
Total fruit[(a)] (g/day)		-17(-29,-5)	0.006	-19 (-34, -3)	0.01
Total veg[(b)] (g/day)		-3 (-16, 9)	0.5	-10 (-26, 6)	0.2
FV portions[(c)]		-0.2 (-0.5, -0.03)	0.09	-0.3 (-0.6, -0.03)	0.08
5-a-day portions[(d)]		-0.2 (-0.5, -0.03)	0.08	-0.3 (-0.5 ,0.05)	0.09
Total FV [(e)](g/day)		-21 (-41, -1)	0.03	-29 (-54, -4)	0.02
**Nutrients (mg)**					
Vitamin C diet only		-1 (-8, 5)	0.7	-5 (-14, 3)	0.2
Vitamin C *		-9 (-26, 8)	0.3	-12 (-34, 8)	0.2
**Biomarkers (μmol/l)**					
Vitamin C	n= 550	-2 (-6, 0.7)	0.1	4 (-0.01, 9)	0.05
Total caro [(f)]	n= 440	-0.2 (-0.4, -0.05)	0.01	-0.04 (-0.2 , 0.2)	0.7
α-carotene	n= 563	-0.002 (-0.01, 0.01)	0.7	-0.002 (-0.02, 0.01)	0.7
β-carotene	n= 588	-0.03 (-0.09 ,0.01)	0.1	0.03 (-0.04, 0.1)	0.4
Lycopene	n= 585	-0.09(-0.1, -0.02)	0.007	-0.01(-0.1, 0.07)	0.7

Physical activity was time spent at moderate or vigorous physical activity (hour/day).

For peer review only

Table 6. The association between weekday/weekend sleep duration categories and FV intakes and associated biomarkers for adults from the NDNS year 1-4.

Weekday sleep categories compared to reference group (7-8 hours/day)				
**Model 2 (n=1610)**	**Short sleepers <7 hours/day**		**Long sleepers >8 hours/day**	
**FV intake**	**Mean difference (95%CI)**	**P value**	**Mean difference (95%CI)**	**P value**
Total fruit [a] (g/day)	-13 (-23, -2)	0.01	-28 (-44, -12)	0.001
Total veg [b] (g/day)	-8 (-19, 2)	0.1	-16 (-32, -0.1)	0.04
FV portions[c]	-0.2 (-0.4,-0.02)	0.03	-0.5 (-0.8, -0.1)	0.003
5-a-day portions[d]	-0.2 (-0.4 , -0.03)	0.02	-0.5 (-0.8, -0.1)	0.004
Total FV[e] (g/day)	-21 (-38, -4)	0.01	-44 (-70, -19)	0.001
**Nutrients (mg)**				
Vitamin C diet only	-3 (-10, 2)	0.2	-8 (-18, 0.9)	0.07
Vitamin C **	-4 (-18, 9)	0.5	-18 (-38, 2)	0.08
**Biomarkers**(μmol/l)				
Vitamin C n=718	-4 (-7, -0.9)	0.01	0.04 (-4, 4)	0.9
Total caro[f] n= 520	-0.2 (-0.4, -0.06)	0.008	-0.09 (-0.3, 0.1)	0.4
α-carotene n=719	-0.005 (-0.01, 0.007)	0.3	-0.004 (-0.02, 0.01)	0.6
β-carotene n= 765	-0.05 (-0.1, -0.01)	0.01	0.01 (-0.06,0.08)	0.7
Lycopene n= 763	-0.08 (-0.1, -0.01)	0.01	-0.08 (-0.1,0.01)	0.09
**Weekend sleep categories compared to reference group (7-8 hours/day)**				
**Model 2 (n=1610)**	**Short sleepers <7 hours/day**		**Long sleepers >8 hours/day**	
**FV intake**	**Mean difference (95%CI)**	**P value**	**Mean difference (95%CI)**	**P value**
Total fruit [a] (g/day)	-10 (-22, 1)	0.07	-3 (-15, 8)	0.5
Total veg [b] (g/day)	-14 (-26, -2)	0.01	-6 (-18, 6)	0.3
FV portions[c]	-0.3 (-0.5 ,-0.05)	0.01	-0.1 (-0.3, 0.1)	0.4
5-a-day portions[d]	-0.2 (-0.5,-0.03)	0.02	-0.04 (-0.3, 0.2)	0.7
Total FV[e] (g/day)	-24 (-43, -6)	0.010	-9 (-29, 9)	0.3
**Nutrients (mg)**				
Vitamin C diet only	-4 (-11, 2)	0.2	4 (-2, 11)	0.2
Vitamin C *	-8 (-23, 6)	0.2	-1 (-17, 13)	0.8
**Biomarkers**(μmol/l)				
Vitamin C n=717	-4 (-7, -0.6)	0.020	1 (-2, 5)	0.3
Total caro[f] n= 519	-0.2 (-0.4, -0.1)	0.003	-0.05 (-0.2, 0.1)	0.6
α-carotene n= 718	-0.004 (-0.01, 0.009)	0.5	-0.001 (-0.01, 0.01)	0.8
β-carotene n= 764	-0.05(-0.1, -0.0006)	0.04	0.01 (-0.04,0.07)	0.6
Lycopene n= 762	-0.09 (-0.1, -0.03)	0.003	-0.05 (-0.1,0.01)	0.1

For peer review only - http://bmjopen.bmj.com/site/about/guidelines.xhtml

For peer review only

Table 7. The association between sleep duration categories and FV intakes, nutrients and associated biomarkers for adults from the NDNS years 1-4 stratified by BMI.

Short sleepers (<7h/d) and Long sleepers (>8h/d) compared to the reference group (7-8 h/d) stratified by BMI												
BMI categories	BMI 18.5-25 n= 524				BMI 25-30 n= 525				BMI ≥30 n= 438			
	Short sleepers <7 h/d		Long sleepers >8 h/d		Short sleepers <7 h/d		Long sleepers >8 h/d		Short sleepers <7 h/d		Long sleepers >8 h/d	
**FV intake Model 2**	**Mean difference (95%CI)**	**P value**	**Mean difference (95%CI)**	**P value**	**Mean difference (95%CI)**	**P value**	**Mean difference (95%CI)**	**P value**	**Mean difference (95%CI)**	**P value**	**Mean difference (95%CI)**	**P Value**
Total fruit[(a)] (g/day)	-22 (-45,-0.4)	0.04	-26 (-50,-1)	0.03	-8 (-26,9)	0.3	-4 (-27,18)	0.7	-16(-36,2)	0.09	-22 (-51,5)	0.1
Total veg[(b)] (g/day)	-23 (-45,-1)	0.04	-11 (-35,12)	0.3	3 (-15,23)	0.6	-3 (-27,21)	0.8	-21(-41,-1)	0.03	-24(-53, 3)	0.09
FV portions[(c)]	-0.6 (-1,0.2)	0.004	-0.4 (-0.8,0.08)	0.1	-0.01 (-0.3,0.3)	0.9	-0.1 (-0.6,0.3)	0.6	-0.3(0.7,0.03)	0.07	-0.4 (-1,0.1)	0.1
5-a-day portions[(d)]	-0.6 (-1,-0.1)	0.006	-0.3 (-0.8,0.1)	0.1	-0.02 (-0.4,0.3)	0.9	-0.09 (-0.6,0.4)	0.6	-0.3(-0.8,0.04)	0.07	-0.3(-0.9, 0.2)	0.2
Total FV[(e)](g/day)	-46 (-81,-11)	0.009	-37 (-75,0.1)	0.05	-4 (-33,24)	0.7	-7 (-45,30)	0.6	-38 (-69,-7)	0.01	-47(-92,-2)	0.03
**Nutrients (mg/d)**												
Vitamin C diet only	-6 (-18,5)	0.2	-2 (-15,10)	0.7	-5 (-17,6)	0.3	2 (-13,17)	0.7	-5 (-18,7)	0.4	-4 (-23,14)	0.6
Vitamin C *	4 (-21,31)	0.7	3 (-24,32)	0.7	-26 (-53,0.1)	0.04	-29 (-63,5)	0.1	-0.1(-23,23)	0.9	-0.4(-34,33)	0.9
**Biomarkers (μmol/l)**												
Vitamin C	-3(-9,2)	0.2	2 (-3,9)	0.4	0.06 (-6,5)	0.9	5(-2,12)	0.2	-4(-9,1)	0.1	2(-5,10)	0.5
Total carot[(f)]	-0.4 (-0.7,-0.1)	0.007	-0.2 (-0.5,0.1)	0.2	-0.2 (-0.4,-0.006)	0.04	-0.1 (-0.4,0.1)	0.2	-0.1(-0.3,0.09)	0.2	-0.08(-0.4,0.2)	0.5
α-carotene	-0.01 (-0.04,0.009)	0.2	-0.01 (-0.04,0.01)	0.4	-0.007 (-0.02, 0.01)	0.4	-0.004 (-0.03,0.02)	0.6	0.001(-0.01,0.02)	0.8	-0.01(-0.04,0.01)	0.3
β-carotene	-0.07 (-0.1, -0.03)	0.1	-0.01 (-0.1, 0.1)	0.7	-0.03 (-0.1,0.03)	0.3	0.04(-0.05,0.1)	0.3	-0.002(-0.06,0.05)	0.9	-0.04(-0.1,0.04)	0.3
Lycopene	-0.1 (-0.2,0.08)	0.2	-0.02(-0.2,0.1)	0.8	-0.02 (-0.1, 0.1)	0.7	-0.03 (-0.2, 0.1)	0.7	0.03(-0.1,0.2)	0.6	0.1(-0.1,0.3)	0.4

For peer review only

For peer review only - http://bmjopen.bmj.com/site/about/guidelines.xhtml

Longstanding illness description

1. Cancer (neoplasm) including lumps, masses, tumours, and growths and benign (non-malignant) lumps and cysts.
2. Diabetes including hyperglycaemia.
3. Other endocrine/ metabolic.
4. Mental illness/anxiety/depression/nerves.
5. Mental handicap.
6. Epilepsy/fits/convulsions.
7. Migraine/ headaches.
8. Other problems of nervous system.
9. Cataract/ poor eye sight/ blindness.
10. Other eye complaints.
11. Poor hearing/deafness.
12. Tinnitus/noises in the ear.
13. Meniere’s disease/ear complaints causing balance problems.
14. Other ear complaints.
15. Stroke/cerebral haemorrhage/cerebral thrombosis.
16. Heart attack/angina.
17. Hypertension/high blood pressure/blood pressure.
18. Other heart problems.
19. Piles/haemorrhoids including Varicose Veins in anus.
20. Varicose veins/phlebitis in lower extremities.
21. Other blood vessels/embolic.
22. Bronchitis/emphysema.
23. Asthma.
24. Hay fever.
25. Other respiratory complaints.
26. Stomach ulcer/ulcer/abdominal hernia/rupture.
27. Other digestive complaints (stomach, liver, pancreas, bile ducts, small intestine).
28. Complaints of bowel/colon (large intestine, caecum, bowel, colon, rectum).
29. Complaints of teeth/mouth/tongue.
30. Kidney complaints.
31. Urinary tract infection.
32. Other bladder problems/incontinence.
33. Reproductive system disorders.
34. Arthritis/rheumatism/fibrosis.
35. Back problems/slipped disc/spine/neck.
36. Other problems of bones/joints/muscles.
37. Infectious and parasitic disease.
38. Disorders of blood and blood forming organs and immunity disorders.
39. Skin complaints.
40. Other complaints.
41. Unclassifiable (no other codable complaint).
42. Complaint no longer present.

For peer review only - http://bmjopen.bmj.com/site/about/guidelines.xhtml

Table 1-7 legends

Model 2 adjusted for age, gender, socio-economic status, smoking, ethnicity and food energy. G, gram, CI, Confidence interval, veg, vegetables, mg, milligram, µmol, micromole, l, litre, n, number, FV, fruits and vegetables, BMI, body mass index

a) Total fruit (not including juice) = Fruit(g)+Dried fruit (g)+ Smoothie fruit (g)
b) Total vegetables= Beans (g) + Brassicaceae (g) + Other Vegg + Tomatoes (g) + Tomato Puree (g) +Yellow Red Green (g).
c) FV portions= (Fruit (g) + Driedfruitx3_mean + Tompureex5 mean + beans max mean+ Brassicaceae (g) + Yellow Red Green (g) + Other veg (g) + Tomatoes (g)) / 80 .
d) 5-a-day portions(portions/day)= Fruit veg portions + Fruit juice portions+ Smoothie Fruit portions
e) Total FV (not including juice) = Total fruit +Total vegetables
f) Total carotenoids = Lutein + alpha-cryptoxanthin + beta-cryptoxanthin+ lycopene + alpha-carotene + beta-carotene
   *Vitamin C including supplements

For peer review only

For peer review only - http://bmjopen.bmj.com/site/about/guidelines.xhtml

STROBE Statement—checklist of items that should be included in reports of observational studies

	Item No	Recommendation	Page
**Title and abstract**	1	(*a*) Indicate the study's design with a commonly used term in the title or the abstract	Pg. 1
		(*b*) Provide in the abstract an informative and balanced summary of what was done and what was found	Pg. 2
**Introduction**			
Background/rationale	2	Explain the scientific background and rationale for the investigation being reported	Pg. 3
Objectives	3	State specific objectives, including any prespecified hypotheses	Pg. 3
**Methods**			
Study design	4	Present key elements of study design early in the paper	Pg. 1
Setting	5	Describe the setting, locations, and relevant dates, including periods of recruitment, exposure, follow-up, and data collection	Pg. 3-5
Participants	6	(*a*) *Cohort study*—Give the eligibility criteria, and the sources and methods of selection of participants. Describe methods of follow-up<br>*Case-control study*—Give the eligibility criteria, and the sources and methods of case ascertainment and control selection. Give the rationale for the choice of cases and controls<br>*Cross-sectional study*—Give the eligibility criteria, and the sources and methods of selection of participants	Fig. 1
		(*b*) *Cohort study*—For matched studies, give matching criteria and number of exposed and unexposed<br>*Case-control study*—For matched studies, give matching criteria and the number of controls per case	NA
Variables	7	Clearly define all outcomes, exposures, predictors, potential confounders, and effect modifiers. Give diagnostic criteria, if applicable	Pg. 4-6
Data sources/ measurement	8*	For each variable of interest, give sources of data and details of methods of assessment (measurement). Describe comparability of assessment methods if there is more than one group	Pg. 4-6
Bias	9	Describe any efforts to address potential sources of bias	NA
Study size	10	Explain how the study size was arrived at	Fig. 1
Quantitative variables	11	Explain how quantitative variables were handled in the analyses. If applicable, describe which groupings were chosen and why	Pg. 5-6
Statistical methods	12	(*a*) Describe all statistical methods, including those used to control for confounding	Pg. 5-6
		(*b*) Describe any methods used to examine subgroups and interactions	NA
		(*c*) Explain how missing data were addressed	Pg. 6
		(*d*) *Cohort study*—If applicable, explain how loss to follow-up was addressed<br>*Case-control study*—If applicable, explain how matching of cases and controls was addressed<br>*Cross-sectional study*—If applicable, describe analytical methods taking account of sampling strategy	Pg. 5
		(*e*) Describe any sensitivity analyses	Pg. 5-6

Continued on next page

For peer review only - http://bmjopen.bmj.com/site/about/guidelines.xhtml

Results			Page
Participants	13*	(a) Report numbers of individuals at each stage of study—eg numbers potentially eligible, examined for eligibility, confirmed eligible, included in the study, completing follow-up, and analysed	Fig. 1
		(b) Give reasons for non-participation at each stage	NA
		© Consider use of a flow diagram	Fig. 1
Descriptive data	14*	(a) Give characteristics of study participants (eg demographic, clinical, social) and information on exposures and potential confounders	Pg. 6-7
		(b) Indicate number of participants with missing data for each variable of interest	NA
		(c) *Cohort study*—Summarise follow-up time (eg, average and total amount)	NA
Outcome data	15*	*Cohort study*—Report numbers of outcome events or summary measures over time	NA
		Case-control study—Report numbers in each exposure category, or summary measures of exposure	NA
		Cross-sectional study—Report numbers of outcome events or summary measures	Pg. 7
Main results	16	(*a*) Give unadjusted estimates and, if applicable, confounder-adjusted estimates and their precision (eg, 95% confidence interval). Make clear which confounders were adjusted for and why they were included	Pg. 8-9
		(*b*) Report category boundaries when continuous variables were categorized	Pg. 5-6
		(*c*) If relevant, consider translating estimates of relative risk into absolute risk for a meaningful time period	NA
Other analyses	17	Report other analyses done—eg analyses of subgroups and interactions, and sensitivity analyses	Pg. 10
**Discussion**			
Key results	18	Summarise key results with reference to study objectives	Pg. 11
Limitations	19	Discuss limitations of the study, taking into account sources of potential bias or imprecision. Discuss both direction and magnitude of any potential bias	Pg.13
Interpretation	20	Give a cautious overall interpretation of results considering objectives, limitations, multiplicity of analyses, results from similar studies, and other relevant evidence	Pg.12-14
Generalisability	21	Discuss the generalisability (external validity) of the study results	Pg. 12-14
**Other information**			
Funding	22	Give the source of funding and the role of the funders for the present study and, if applicable, for the original study on which the present article is based	Pg.15

*Give information separately for cases and controls in case-control studies and, if applicable, for exposed and unexposed groups in cohort and cross-sectional studies.

Note: An Explanation and Elaboration article discusses each checklist item and gives methodological background and published examples of transparent reporting. The STROBE checklist is best used in conjunction with this article (freely available on the Web sites of PLoS Medicine at http://www.plosmedicine.org/, Annals of Internal Medicine at http://www.annals.org/, and Epidemiology at http://www.epidem.com/). Information on the STROBE Initiative is available at www.strobe-statement.org.

For peer review only
